# Caspase‐11 counteracts mitochondrial ROS‐mediated clearance of *Staphylococcus aureus* in macrophages

**DOI:** 10.15252/embr.201948109

**Published:** 2019-10-21

**Authors:** Kathrin Krause, Kylene Daily, Shady Estfanous, Kaitlin Hamilton, Asmaa Badr, Arwa Abu Khweek, Rana Hegazi, Midhun NK Anne, Brett Klamer, Xiaoli Zhang, Mikhail A Gavrilin, Vijay Pancholi, Amal O Amer

**Affiliations:** ^1^ Department of Microbial Infection and Immunity Infectious Diseases Institute Ohio State University Columbus OH USA; ^2^ Department of Biology and Biochemistry Birzeit University Birzeit West Bank Palestine; ^3^ Center for Biostatistics Ohio State University Columbus OH USA; ^4^ Department of Internal Medicine Ohio State University Columbus OH USA; ^5^ Department of Pathology Ohio State University Columbus OH USA

**Keywords:** caspase‐11, macrophages, mitochondria, ROS, *Staphylococcus aureus*, Autophagy & Cell Death, Immunology, Microbiology, Virology & Host Pathogen Interaction

## Abstract

Methicillin‐resistant *Staphylococcus aureus* (MRSA) is a growing health concern due to increasing resistance to antibiotics. As a facultative intracellular pathogen, MRSA is capable of persisting within professional phagocytes including macrophages. Here, we identify a role for CASP11 in facilitating MRSA survival within murine macrophages. We show that MRSA actively prevents the recruitment of mitochondria to the vicinity of the vacuoles they reside in to avoid intracellular demise. This process requires CASP11 since its deficiency allows increased association of MRSA‐containing vacuoles with mitochondria. The induction of mitochondrial superoxide by antimycin A (Ant A) improves MRSA eradication in *casp11* ^−/−^ cells, where mitochondria remain in the vicinity of the bacterium. In WT macrophages, Ant A does not affect MRSA persistence. When mitochondrial dissociation is prevented by the actin depolymerizing agent cytochalasin D, Ant A effectively reduces MRSA numbers. Moreover, the absence of CASP11 leads to reduced cleavage of CASP1, IL‐1β, and CASP7, as well as to reduced production of CXCL1/KC. Our study provides a new role for CASP11 in promoting the persistence of Gram‐positive bacteria.

## Graphical abstract

*Staphylococcus aureus* exploits caspase‐11 to dissociate MRSA‐containing vacuoles from mitochondria. Caspase‐11 also promotes inflammasome activation and secretion of IL‐1α, IL‐1β, and CXCL1/KC in response to MRSA infection in macrophages.

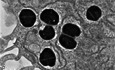

## Introduction

Methicillin‐resistant *Staphylococcus aureus* (MRSA) refers to a group of Gram‐positive cocci that have developed a resistance to most β‐lactam antibiotics due to the expression of a penicillin‐binding protein (PBP2a) 1. As an opportunistic pathogen, *S. aureus* exhibits a broad repertoire of virulence factors and can cause a variety of clinical manifestations, ranging from localized mild skin and soft tissue infections to severe invasive diseases with potentially fatal outcomes such as pneumonia, endocarditis, and sepsis 2, 3. Genetically diverse MRSA isolates can be found in healthcare facilities as well as communities all over the world, and resistances against antibiotics of last resort, such as vancomycin, have emerged 4. Alternative treatment strategies are therefore necessary to overcome multidrug‐resistant MRSA infections.

Inflammatory caspase‐11/caspase‐4 (CASP11) contributes to non‐canonical NLRP3 inflammasome activation and subsequent inflammation 5. CASP11 is not expressed in healthy tissue unless induced by infection or other pathologic stress 6, 7, 8, 9. Until recently, appreciated functions of CASP11 were the recognition of cytosolic LPS followed by the activation of CASP1, cleavage of gasdermin D (GSDMD), pro‐inflammatory cytokine secretion, and cell death 5, 9, 10, 11. Additionally, the role of CASP11 is dependent on the infectious agent. While CASP11 deficiency has been shown to protect mice from LPS‐induced endotoxemia due to reduced release of the inflammatory mediators IL‐1α, IL‐1β, and CXCL1/KC 5, 9, 12, the absence of CASP11 in the context of Gram‐negative bacterial infections promotes bacterial replication and dissemination in mice 8, 9, 13, 14. Furthermore, CASP11 was shown to modulate the intracellular trafficking of pathogens, such as *L. pneumophila* and *B. cenocepacia,* leading to their degradation within lysosomes 8, 9, 13. In contrast, little is known about the role of CASP11 in the immune defense against Gram‐positive bacteria. Recently, purified lipoteichoic acid (LTA), a cell wall component from Gram‐positive bacteria, was reported to induce CASP11 activity via NLRP6 15. However, unlike mice infected with Gram‐negative bacteria, mice deficient of CASP11 exhibit improved survival and efficient bacterial clearance in response to Gram‐positive pathogens such as *Listeria monocytogenes* and *S. aureus* 15. The study by Hara *et al* demonstrated that increased production of IL‐18 in WT mice impairs clearance of *L. monocytogenes*. However, during *S. aureus* infection, others have shown that the neutralization of IL‐1β or IL‐18 does not influence survival or pulmonary burdens of mice 16. Therefore, the mechanism behind reduced susceptibility of *casp11* ^−/−^ mice to Gram‐positive bacteria is not completely understood and may vary according to the pathogen.

Mitochondria are the main source for cellular ATP production. While mitochondrial reactive oxygen species (mtROS), such as superoxide and hydrogen peroxide, are generally considered byproducts of oxidative phosphorylation at the inner mitochondrial membrane, there is increasing evidence that mtROS can also augment the bactericidal capacity of macrophages 17, 18. The recruitment of mitochondria to phagosomes containing intracellular bacteria and subsequent elevated mtROS production have been shown to mediate the antibacterial response against *Salmonella enterica* serovar Typhimurium 17. Likewise, TNF‐induced mtROS facilitate clearance of *Mycobacterium tuberculosis* from macrophages 19. Co‐localization of internalized *S. aureus* with mitochondria was documented for both α‐hemolysin (Hla)‐deficient strains and in response to chemical inhibition of NLRP3, resulting in bacterial clearance by mtROS 16. Here, we propose a role for CASP11 in facilitating MRSA evasion from mtROS‐mediated killing. We report that CASP11 deficiency leads to an increased association of MRSA with mitochondria, which is accompanied by elevated mtROS production and decreased inflammasome activation, thereby promoting more efficient clearance from murine macrophages. Antimycin A (Ant A) treatment, which inhibits complex III of the electron transport chain (ETC) thus raising mitochondrial superoxide production, further improves the bactericidal capacity of *casp11* ^−/−^ macrophages against MRSA. In WT macrophages, the inhibition of the actin cytoskeleton via cytochalasin D (Cyto D) prevents the dissociation of phagocytosed MRSA from mitochondria and hence restores Ant A‐induced bacterial killing. Together, these results provide a novel role for CASP11 in the pathogenesis of Gram‐positive bacteria.

## Results

### CASP11 contributes to MRSA‐induced inflammasome activation in murine macrophages

**Figure 1:**
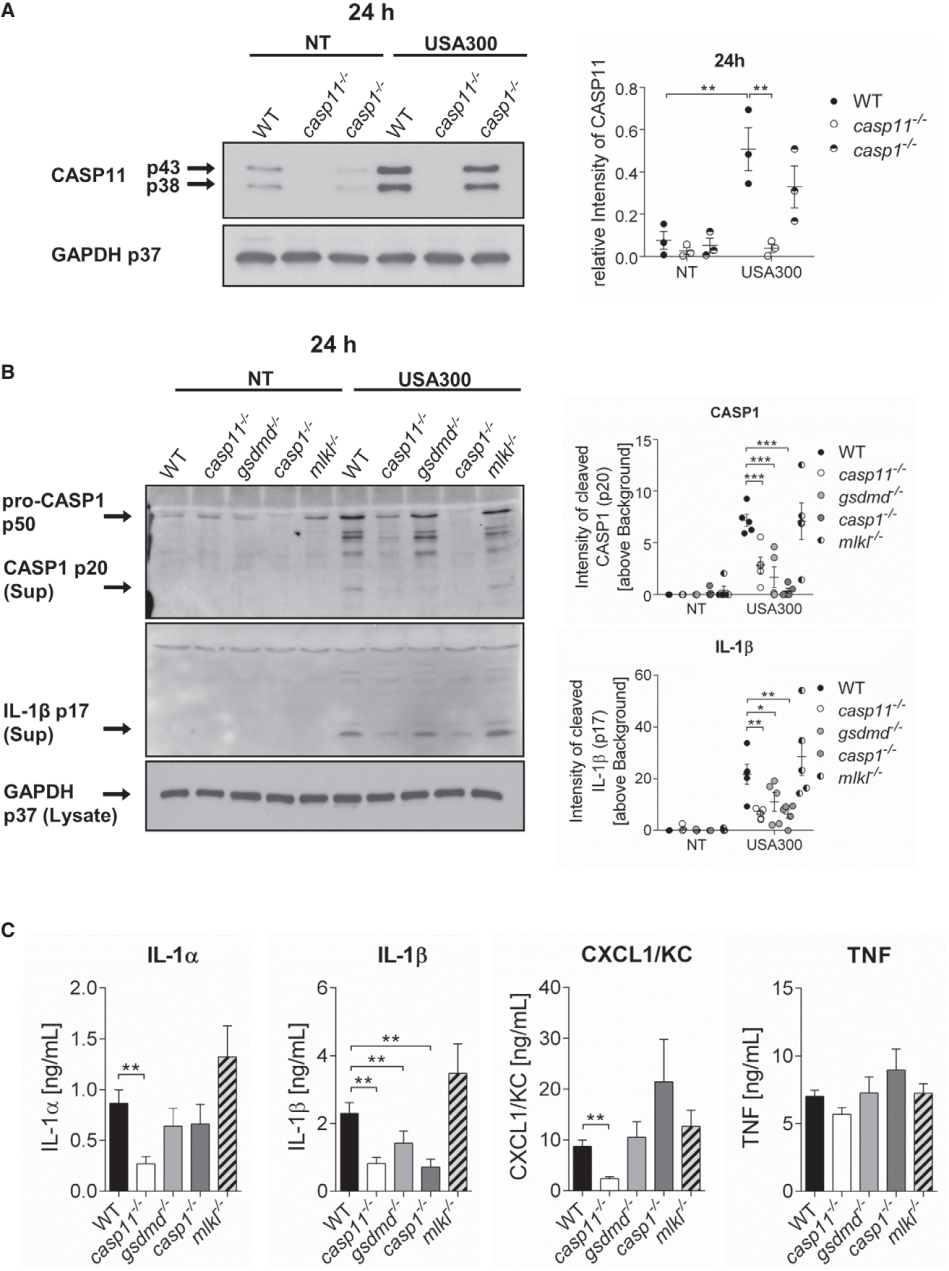
CASP11 contributes to MRSA‐induced inflammasome activation in murine macrophages Immunoblot analysis of CASP11 from WT, *casp11* ^−/−^, and *casp1* ^−/−^ BMDMs infected with MRSA (MOI 5:1) at 24 h post‐infection. Densitometry analysis represents mean ± SEM (*n* = 3 biological replicates). Statistical analysis was performed using two‐way ANOVA. **P ≤ 0.01, NT = no treatment.Immunoblot analysis of cleaved CASP1 and IL‐1β in supernatants from WT, *casp11* ^−/−^, *gsdmd* ^−/−^, *casp1* ^−/−^, and *mlkl* ^−/−^ BMDMs infected with MRSA (MOI 20:1) at 24 h post‐infection. Densitometry analysis represents mean ± SEM (*n* = 5 biological replicates). Statistical analysis was performed using two‐way ANOVA. *P ≤ 0.05, **P ≤ 0.01, ***P ≤ 0.001, NT = no treatment.Cytokine release from WT, *casp11* ^−/−^, *gsdmd* ^−/−^, *casp1* ^−/−^, and *mlkl* ^−/−^ BMDMs infected with MRSA (MOI 20:1) at 24 h post‐infection. Data represent mean ± SEM (*n* = 8 biological replicates). Statistical analysis was performed using one‐way ANOVA. **P ≤ 0.01.

**Figure EV1:**
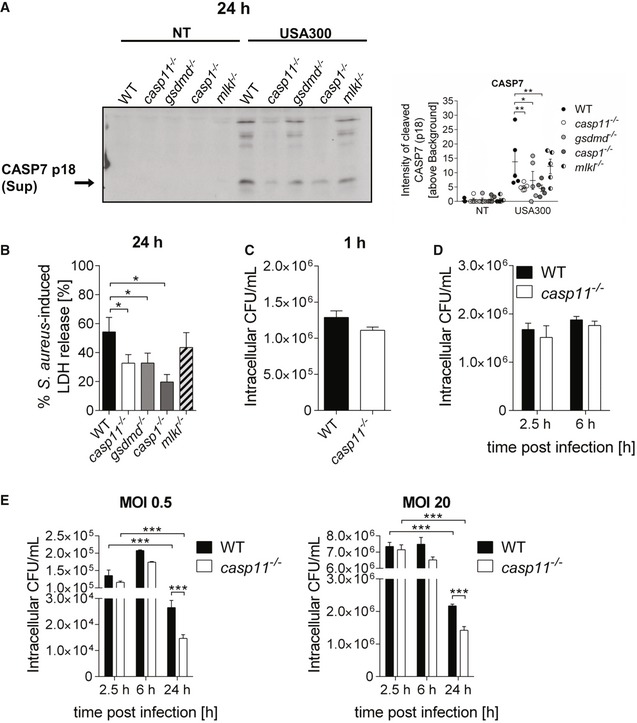
CASP11 promotes MRSA‐induced inflammasome activation and MRSA persistence in macrophages Immunoblot analysis of cleaved CASP7 in supernatants from WT, *casp11* ^−/−^, *gsdmd* ^−/−^, *casp1* ^−/−^, and *mlkl* ^−/−^ BMDMs infected with MRSA (MOI 20:1) at 24 h post‐infection. Densitometry analysis represents mean ± SEM (*n* = 5 biological replicates). Statistical analysis was performed using two‐way ANOVA. *P ≤ 0.05, **P ≤ 0.01, NT = no treatment.MRSA‐induced LDH released in supernatants from WT, *casp11* ^−/−^, *gsdmd* ^−/−^, *casp1* ^−/−^, and *mlkl* ^−/−^ BMDMs at 24 h post‐infection (MOI 20:1). Data represent mean ± SEM (*n* = 8 biological replicates). Statistical analysis was performed using one‐way ANOVA. *P ≤ 0.05.Intracellular CFU of MRSA (MOI 5:1) in WT and *casp11* ^−/−^ BMDMs at 1 h post‐infection. Data represent mean ± SEM (*n* = 3 biological replicates). Statistical analysis was performed using two‐tailed Student's *t*‐test.Intracellular survival of MRSA (MOI 5:1) in WT and *casp11* ^−/−^ BMDMs. Data represent mean ± SEM (*n* = 5 biological replicates). Statistical analysis was performed using a linear mixed effects model.Intracellular survival of MRSA (MOI 0.5:1 and 20:1) in WT and *casp11* ^−/−^ BMDMs. Data represent mean ± SEM (*n* = 3 biological replicates). Statistical analysis was performed using a linear mixed effects model. ***P ≤ 0.001.

*Staphylococcus aureus* activates CASP1 through the NLRP3 inflammasome, leading to the secretion of IL‐1β and cell death 20, 21, 22, 23. While CASP11 was long believed to solely recognize cytosolic LPS from Gram‐negative bacteria, leading to non‐canonical NLRP3 inflammasome activation 6, 7, LTA derived from Gram‐positive bacteria has been shown to promote CASP11 cleavage and activation 15. Since resting cells exhibit low levels of CASP11, we infected bone marrow‐derived macrophages (BMDMs) from WT, *casp11* ^−/−^, and *casp1* ^−/−^ mice with the MRSA strain USA300 at MOI 5:1 to determine whether intracellular MRSA stimulates CASP11 protein expression. At 24 h post‐infection, we found a significant up‐regulation of CASP11 in cell lysates of infected WT as well as *casp1* ^−/−^ macrophages (Fig 1A). To further elucidate whether CASP11 plays a role in inflammasome activation in response to MRSA*,* we evaluated cleavage of CASP1 and IL‐1β in cell culture supernatants from WT and *casp11* ^−/−^ macrophages via Western blot analysis at 24 h post‐infection (MOI 20:1). BMDMs from *casp1* ^−/−^ and *gsdmd* ^−/−^ mice were included to serve as a control for absent CASP1 or IL‐1β secretion, respectively. In addition, we also added BMDMs deficient of the pseudokinase MLKL, which is a known effector protein for the necroptosis pathway. The inhibition of MLKL was demonstrated to reduce MRSA‐induced IL‐1β secretion from THP‐1 macrophages 24, suggesting potential crosstalk between pyroptosis and necroptosis during MRSA infection. Compared to corresponding WT cells, cleavage and secretion of CASP1 and IL‐1β were significantly reduced in supernatants from MRSA‐infected *casp11* ^−/−^, *gsdmd* ^−/−^, and *casp1* ^−/−^ but not *mlkl* ^−/−^ macrophages (Fig 1B and C). Since CASP1 has been shown to activate CASP7 25, 26, we also analyzed the processing of CASP7 in response to MRSA. Similar to CASP1 and IL‐1β, we found significantly lower amounts of CASP7 cleavage products in the supernatants from infected *casp11* ^−/−^, *gsdmd* ^−/−^, and *casp1* ^−/−^ but not *mlkl* ^−/−^ macrophages (Fig EV1A). In accordance with decreased secretion of CASP1, IL‐1β, and CASP7, there were also lower amounts of the enzyme lactate dehydrogenase (LDH) in cell culture supernatants from MRSA‐infected *casp11* ^−/−^, *gsdmd* ^−/−^, and *casp1* ^−/−^ macrophages when compared to WT or *mlkl* ^−/−^ cells (Fig EV1B). Interestingly, *gsdmd* ^−/−^ macrophages demonstrated mildly increased levels of secreted IL‐1β and CASP7 compared to their *casp11* ^−/−^ and *casp1* ^−/−^ counterparts (Fig 1B), suggesting GSDMD‐independent mechanisms contributing to the secretion of cleaved IL‐1β and CASP7. Reduced secretion of IL‐1α and CXCL1/KC was found only in *casp11* ^−/−^ macrophages (Fig 1C). We previously reported decreased CXCL1/KC production in *casp11* ^−/−^ macrophages and mice in response to *B. cenocepacia* infection 9. Here, our data with MRSA support the idea of a general defect in CXCL1/KC production in the absence of CASP11. No difference in the secretion of the inflammasome independent cytokine TNF could be observed among all five macrophage genotypes. Together, these results demonstrate that CASP11 but not MLKL contributes to MRSA‐induced activation of CASP1 and subsequent processing of IL‐1β and CASP7 in murine macrophages.

### Human CASP4 and CASP5, homologs of murine CASP11, are involved in IL‐1β secretion and cell death induced by MRSA in THP‐1 macrophages

**Figure 2:**
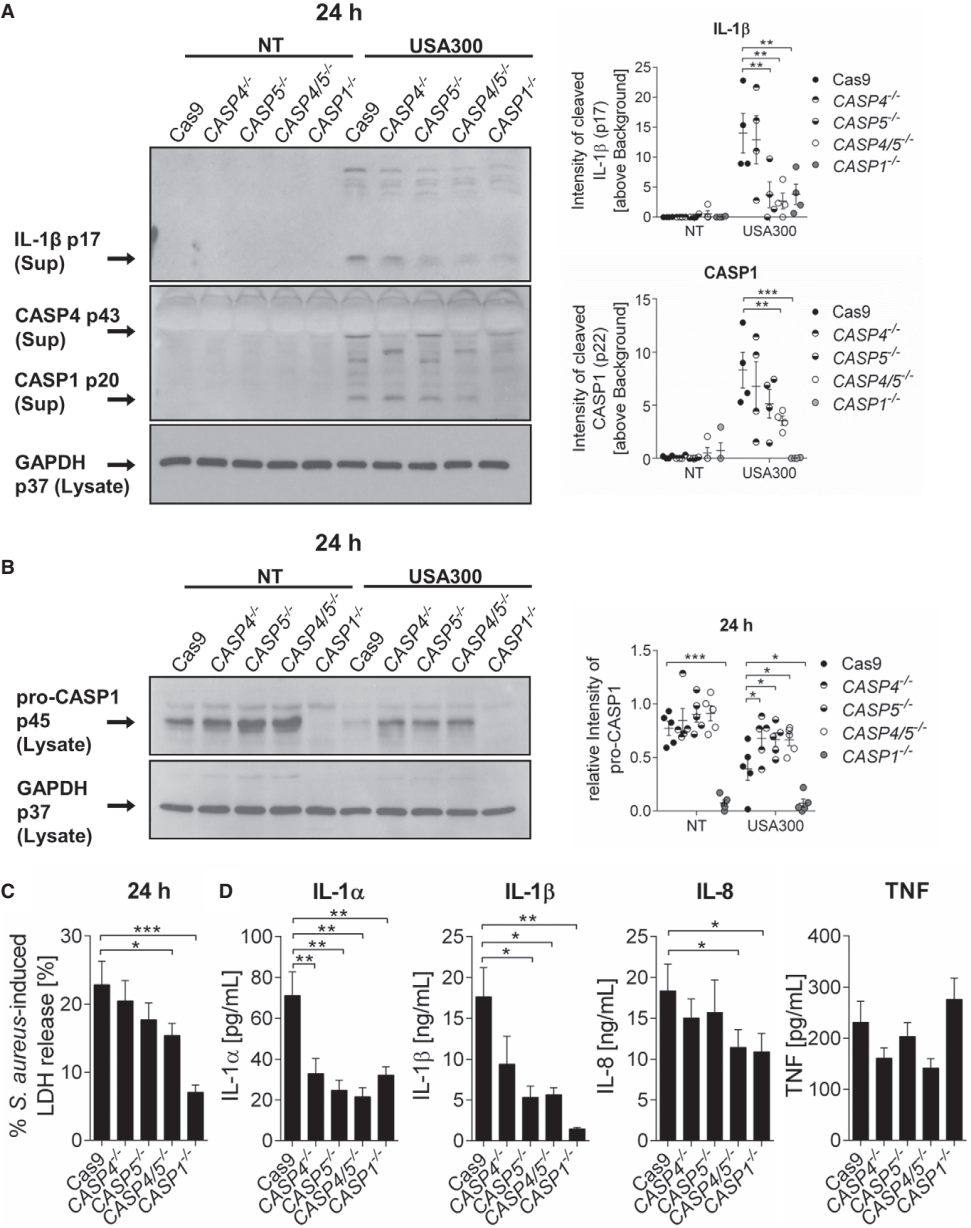
CASP4/5 mediate MRSA‐induced CASP1 activation and IL‐1β release in human THP‐1 macrophages Immunoblot analysis of cleaved IL‐1β and CASP1 (p20) in supernatants from Cas9 control, *CASP4* ^−/−^, *CASP5* ^−/−^, *CASP4/5* ^−/−^, and *CASP1* ^−/−^ THP‐1 cells infected with MRSA at 24 h post‐infection. Densitometry analysis represents mean ± SEM (*n* = 4 biological replicates). Statistical analysis was performed using two‐way ANOVA. **P ≤ 0.01, ***P ≤ 0.001, NT = no treatment.Immunoblot analysis of pro‐CASP1 (p45) in lysates from Cas9 control, *CASP4* ^−/−^, *CASP5* ^−/−^, *CASP4/5* ^−/−^, and *CASP1* ^−/−^ THP‐1 cells infected with MRSA at 24 h post‐infection. Densitometry analysis represents mean ± SEM (*n* = 5 biological replicates). Statistical analysis was performed using two‐way ANOVA. *P ≤ 0.05, ***P ≤ 0.001, NT = no treatment.MRSA‐induced LDH released in supernatants from Cas9 control, *CASP4* ^−/−^, *CASP5* ^−/−^, *CASP4/5* ^−/−^, and *CASP1* ^−/−^ THP‐1 cells at 24 h post‐infection. Data represent mean ± SEM (*n* = 15 biological replicates). Statistical analysis was performed using one‐way ANOVA. *P ≤ 0.05, ***P ≤ 0.001.Cytokine release from Cas9 control, *CASP4* ^−/−^, *CASP5* ^−/−^, *CASP4/5* ^−/−^, and *CASP1* ^−/−^ THP‐1 cells infected with MRSA at 24 h post‐infection. Data represent mean ± SEM (*n* = 10 biological replicates). Statistical analysis was performed using one‐way ANOVA. *P ≤ 0.05, **P ≤ 0.01.

To determine whether CASP4 and CASP5, the human homologs of murine CASP11, contribute to MRSA‐induced inflammasome activity in human THP‐1 macrophages, we used corresponding knockout cells generated using the CRISPR/Cas system (a generous gift of Dr. Seth Masters, Walter and Eliza Hall Institute of Medical Research, Australia) 27. Single *CASP1* ^−/−^ and Cas9 THP‐1 cells (from Dr. Masters) were included as controls. The CRISPR system was induced by incubating the cells with doxycycline. Then, THP‐1 cells were differentiated into macrophages using phorbol 12‐myristate 13‐acetate (PMA) and infected with MRSA at MOI 5:1 for 24 h. As compared to Cas9 control cells, cleavage products of IL‐1β and CASP1 were significantly reduced in the supernatants of infected *CASP4/5* ^−/−^ cells (Fig 2A). *CASP4/5* ^−/−^ cells also exhibited reduced processing of pro‐CASP1 in cell lysates (Fig 2B) and a decreased release of LDH in response to MRSA (Fig 2C). In addition, the absence of both CASP4 and CASP5 resulted in lower amounts of IL‐1α and IL‐8 (human homolog of murine CXCL1/KC) in the supernatants of infected cells (Fig 2D). Similar to murine macrophages, we observed no difference in the release of TNF among all 5 genotypes. These results thus indicate that, similar to murine CASP11, both human CASP4 and CASP5 contribute to MRSA‐induced cleavage of CASP1 and IL‐1β and subsequent cell death.

### CASP11 deficiency improves clearance of intracellular MRSA

**Figure 3:**
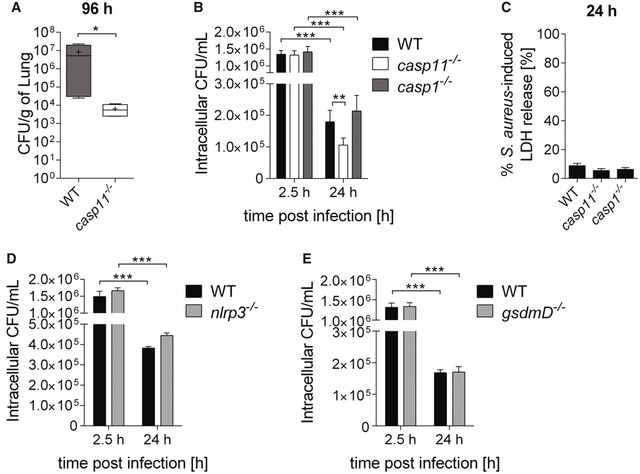
CASP11 deficiency promotes MRSA clearance *in vitro* and *in vivo* *In vivo* CFUs from lungs of WT and *casp11* ^−/−^ mice intratracheally infected with MRSA (2.5 × 10^8^ CFUs/mouse) at 96 h post‐infection (*n* = 4 biological replicates). Boxplot with whiskers from minimum to maximum. Horizontal bands represent the median, and “+” represents the mean. Statistical analysis was performed using two‐tailed Student's *t*‐test. *P ≤ 0.05.Intracellular survival of MRSA in WT, *casp11* ^−/−^, and *casp1* ^−/−^ BMDMs (MOI 5:1). Data represent mean ± SEM (*n* = 12 biological replicates). Statistical analysis was performed using a linear mixed effects model. **P ≤ 0.01, ***P ≤ 0.001.MRSA‐induced LDH release in supernatants from WT, *casp11* ^−/−^, and *casp1* ^−/−^ BMDMs (MOI 5:1) at 24 h post‐infection. Data represent mean ± SEM (*n* = 6 biological replicates). Statistical analysis was performed using one‐way ANOVA.Intracellular survival of MRSA in WT and *nlrp3* ^−/−^ BMDMs (MOI 5:1). Data represent mean ± SEM (*n* = 3 biological replicates). Statistical analysis was performed using a linear mixed effects model. ***P ≤ 0.001.Intracellular survival of MRSA in WT and *gsdmd* ^−/−^ BMDMs (MOI 5:1). Data represent mean ± SEM (*n* = 6 biological replicates). Statistical analysis was performed using a linear mixed effects model. ***P ≤ 0.001.

Although earlier reports indicated that CASP11 has no role in protection against *L. monocytogenes* infection 28, *casp11* ^−/−^ mice were recently demonstrated to have improved survival rates and lower bacterial loads in response to intravenously delivered *L. monocytogenes* as well as *S. aureus* 8325‐4 15, indicating that the absence of CASP11 helps with the clearance of some Gram‐positive bacteria. To investigate the impact of CASP11 on bacterial pulmonary loads, we intratracheally infected WT and *casp11* ^−/−^ mice with 2.5 × 10^8^ CFU of MRSA and determined the bacterial burden at 96 h post‐infection. We found significantly reduced numbers of MRSA in the lungs of *casp11* ^−/−^ mice as compared to corresponding WT mice (Fig 3A). Since MRSA was also shown to invade and persist in murine and human macrophages 29, 30, we next elucidated the role of CASP11 for the intracellular survival of MRSA. BMDMs from WT, *casp11* ^−/−^, and *casp1* ^−/−^ mice were infected with MRSA, and intracellular bacterial numbers were determined at 2.5 and 24 h post‐infection. As shown in Fig 3B, the intracellular load of MRSA was significantly lower at 24 h compared to 2.5 h for all three genotypes, indicating bacterial clearance. However, compared to WT or *casp1* ^−/−^ cells, the reduction of intracellular MRSA was more pronounced in macrophages lacking CASP11. LDH release was similar between all three genotypes (Fig 3C). No difference in the intracellular burden of MRSA in WT and *casp11* ^−/−^ macrophages could be observed at earlier time points (Fig EV1C and D). Testing different MOIs of 0.5:1 and 20:1 revealed increased restriction of MRSA at 24 h post‐infection in the absence of CASP11 as well (Fig EV1E). In contrast, while the inhibition of NLRP3 was reported to promote clearance of MRSA from human macrophages 16, we did not find improved killing of MRSA in *nlrp3* ^−/−^ BMDMs (Fig 3D). Since GSDMD is directly cleaved by CASP11, we also determined intracellular survival of MRSA in *gsdmd* ^−/−^ macrophages. Yet, no difference in intracellular bacterial numbers could be found between WT and *gsdmd* ^−/−^ macrophages (Fig 3E). Together, these *in vivo* and *in vitro* data suggest that the macrophage response against MRSA is more effective in the absence of CASP11.

### CASP11 deficiency facilitates MRSA clearance through mtROS

**Figure 4:**
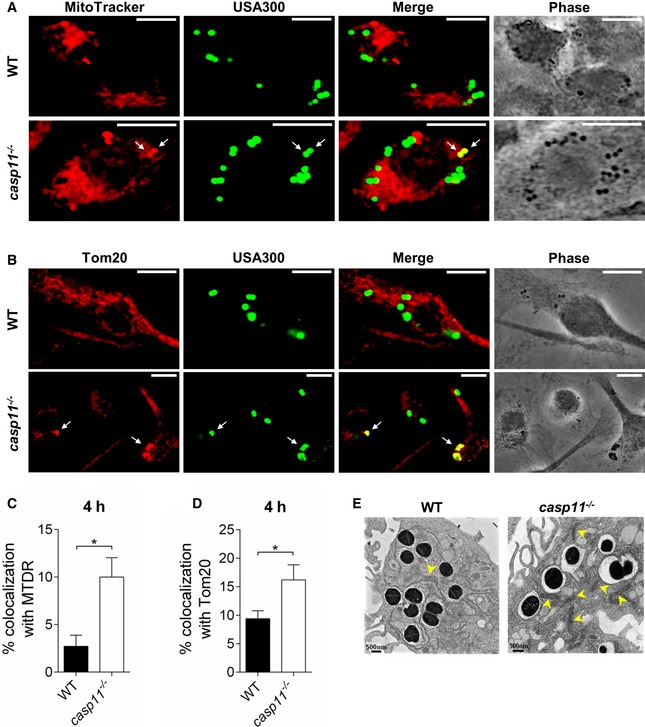
Association of the MRSA‐containing vacuole with mitochondria is increased in *casp11* ^−/−^ macrophages MitoTracker Deep Red immunofluorescence assay of MRSA‐infected WT and *casp11* ^−/−^ BMDMs at 4 h post‐infection. White arrows indicate co‐localization of MRSA with MitoTracker.Tom20 immunofluorescence assay of MRSA‐infected WT and *casp11* ^−/−^ BMDMs at 4 h post‐infection. White arrows indicate co‐localization of MRSA with Tom20.Quantification of MRSA co‐localized with MitoTracker Deep Red. Data represent mean ± SEM (*n* = 3 biological replicates). Statistical analysis was performed using two‐tailed Student's *t*‐test. *P ≤ 0.05.Quantification of MRSA co‐localized with Tom20. Data represent mean ± SEM (*n* = 3 biological replicates). Statistical analysis was performed using two‐tailed Student's *t*‐test. *P ≤ 0.05.Qualitative transmission electron microscopy images of MRSA‐infected WT and *casp11* ^−/−^ macrophages at 4 h post‐infection. Yellow arrows indicate mitochondria. Data information: Scale bars, 10 μm.

**Figure EV2:**
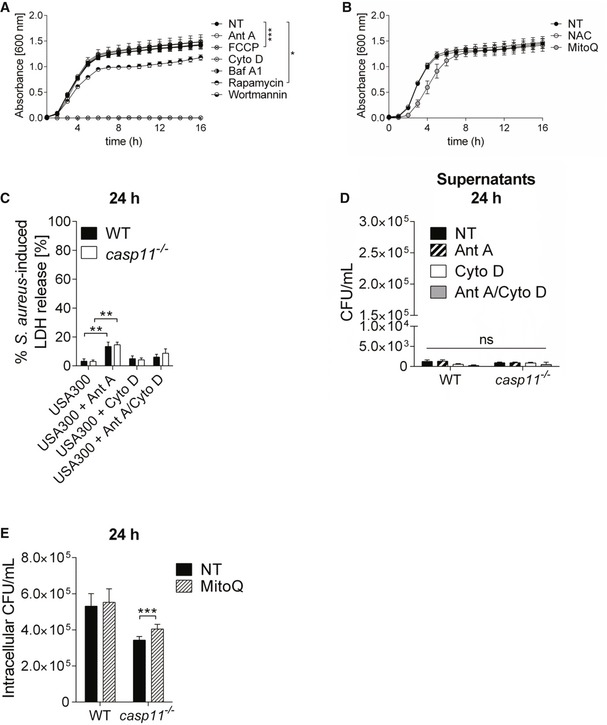
Role of inhibitors affecting mtROS production, autophagy, and F‐actin polymerization on MRSA growth and macrophage viability MRSA growth in TSB in the presence of Ant A, FCCP, Cyto D, BafA1, rapamycin, and wortmannin (*n* = 3 biological replicates). Statistical analysis was performed using two‐way ANOVA. *P ≤ 0.05, ***P ≤ 0.001, NT = no treatment.MRSA growth in TSB in the presence of NAC and MitoQ (*n* = 9 biological replicates). Statistical analysis was performed using two‐way ANOVA. NT = no treatment.MRSA‐induced LDH release in supernatants from WT and *casp11* ^−/−^ BMDMs (MOI 5:1) treated with Ant A or Cyto D at 24 h post‐infection (*n* = 8 biological replicates). Statistical analysis was performed using two‐way ANOVA. **P ≤ 0.01.Extracellular growth of MRSA in cell culture supernatants from WT and *casp11* ^−/−^ BMDMs (MOI 5:1) treated with Ant A or Cyto D at 24 h post‐infection (*n* = 8 biological replicates). Statistical analysis was performed using two‐way ANOVA. NT = no treatment.Intracellular CFU of MRSA in WT and *casp11* ^−/−^ macrophages treated with MitoQ at 24 h post‐infection (MOI 5:1). Data represent mean ± SEM (*n* = 8 biological replicates). Statistical analysis was performed using two‐way ANOVA. ***P ≤ 0.001, NT = no treatment.

**Figure 5:**
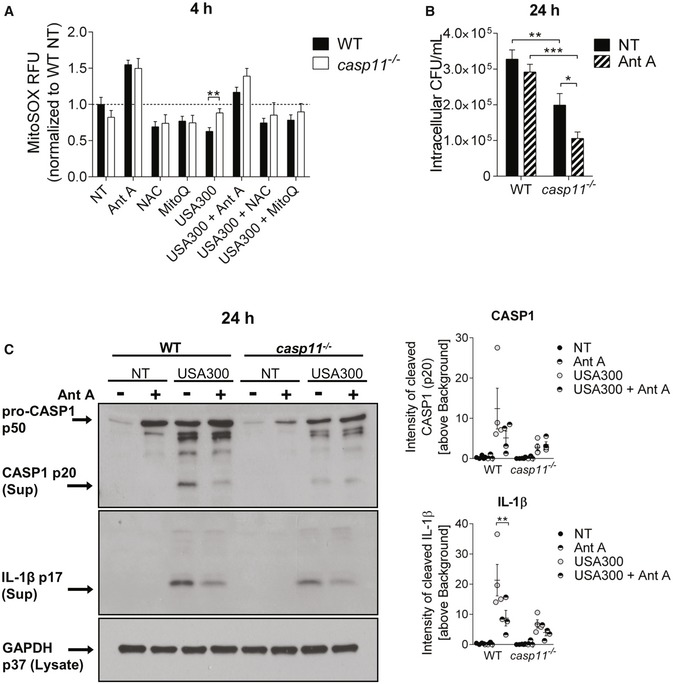
mtROS contributes to MRSA clearance in *casp11* ^−/−^ macrophages MitoSOX assay in WT and *casp11* ^−/−^ BMDMs (MOI 5:1) at 4 h post‐infection. Data represent mean ± SEM (*n* = 15 biological replicates). Statistical analysis was performed using multiple *t*‐tests. **P ≤ 0.01, NT = no treatment.Intracellular CFU of MRSA (MOI 5:1) in WT and *casp11* ^−/−^ BMDMs treated with Ant A at 24 h post‐infection. Data represent mean ± SEM (*n* = 6 biological replicates). Statistical analysis was performed using two‐way ANOVA. *P ≤ 0.05, **P ≤ 0.01, ***P ≤ 0.001, NT = no treatment.Immunoblot analysis of cleaved CASP1 and IL‐1β in supernatants from WT and *casp11* ^−/−^ BMDMs treated with Ant A at 24 h post‐infection (MOI 20:1). Densitometry analysis represents mean ± SEM (*n* = 4 biological replicates). Statistical analysis was performed using two‐way ANOVA. **P ≤ 0.01, NT = no treatment.

It has been shown that mitochondrial ROS production drives the bactericidal activity of macrophages against MRSA 31 and that Hla‐induced NLRP3 inflammasome activation negatively affects the association of phagosomes containing MRSA with mitochondria 16. To test whether mitochondria play a role in the clearance of MRSA in the absence of CASP11, we analyzed co‐localization events between MRSA and mitochondria either stained with MitoTracker Deep Red or an anti‐Tom20 antibody in WT and *casp11* ^−/−^ macrophages. As indicated in Fig 4A–D, *casp11* ^−/−^ macrophages display a higher percentage of bacteria co‐localized with MitoTracker or Tom20 than corresponding WT cells. Electron microscopy revealed bacteria in proximity to mitochondria in *casp11* ^−/−^ but not WT macrophages (Fig 4E). Additionally, to elucidate whether only physical proximity to the mitochondria allows bacterial killing or whether mtROS production itself is increased in *casp11* ^−/−^ macrophages, we measured mitochondrial superoxide production using the MitoSOX Red reagent. Ant A, an inhibitor of complex III of the mitochondrial ETC, served as positive control for superoxide induction, and either the general antioxidant N‐acetylcysteine (NAC) or the mtROS‐quenching agent MitoQ was used to inhibit mtROS production. The chemicals had no direct bactericidal activity against MRSA (Fig EV2A and B). Compared to non‐infected control cells, MRSA suppressed superoxide levels in WT macrophages (Fig 5A). Macrophages lacking CASP11 exhibited higher MitoSOX fluorescence in response to MRSA than corresponding WT cells. The addition of Ant A led to a marked increase in MitoSOX fluorescence in both non‐infected and MRSA‐infected cells. These results suggest that mtROS production was increased in the absence of CASP11. To determine whether increased mtROS production triggered by Ant A further improves bacterial clearance of intracellular MRSA, we treated MRSA‐infected WT and *casp11* ^−/−^ macrophages with Ant A for 24 h and enumerated intracellular bacterial numbers. In contrast to WT cells, wherein no effect could be detected, Ant A treatment significantly reduced the burden of intracellular MRSA in *casp11* ^−/−^ macrophages (Fig 5B). LDH release was significantly increased in response to Ant A, yet no difference in Ant A‐induced cytotoxicity could be found between WT and *casp11* ^−/−^ macrophages (Fig EV2C). There was also no increased extracellular bacterial growth in the presence of Ant A, indicating that gentamicin exclusion was still effective (Fig EV2D). In addition, treatment with the mtROS scavenger MitoQ elevated bacterial numbers in *casp11* ^−/−^ but not WT macrophages (Fig EV2E). This suggests that enhanced proximity to mitochondria contributes to MRSA elimination in response to Ant A‐induced mitochondrial superoxide generation in *casp11* ^−/−^ macrophages.

**Figure EV3:**
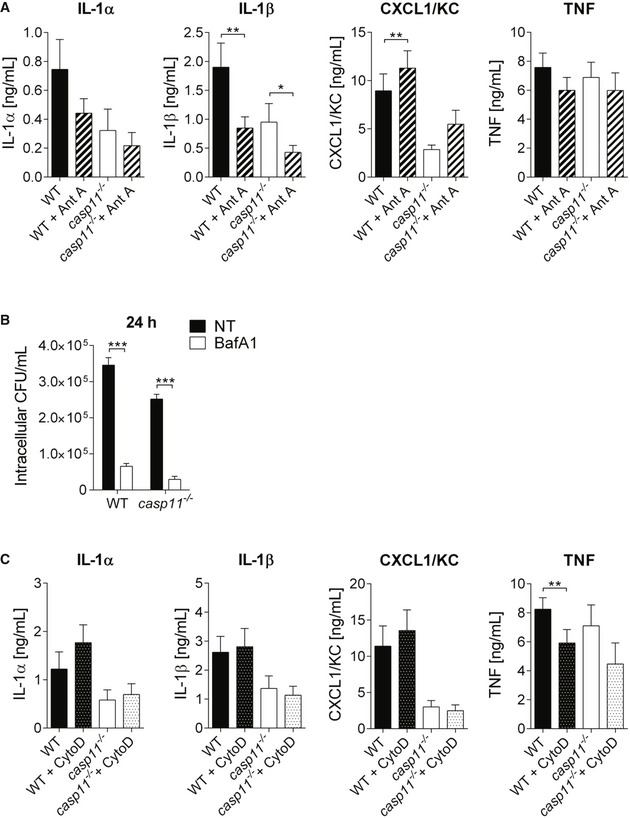
Effects of Ant A, Baf A1, and Cyto D during MRSA infection in macrophages Cytokine release from MRSA‐infected WT and *casp11* ^−/−^ macrophages treated with Ant A at 24 h post‐infection (MOI 20:1). Data represent mean ± SEM (*n* = 8 biological replicates). Statistical analysis was performed using one‐way ANOVA. *P ≤ 0.05, **P ≤ 0.01.Intracellular CFU of MRSA in WT and *casp11* ^−/−^ macrophages treated with BafA1 at 24 h post‐infection (MOI 5:1). Data represent mean ± SEM (*n* = 3 biological replicates). Statistical analysis was performed using two‐way ANOVA. ***P ≤ 0.001, NT = no treatment.Cytokine release from MRSA‐infected WT and *casp11* ^−/−^ macrophages treated with Cyto D at 24 h post‐infection (MOI 20:1). Data represent mean ± SEM (*n* = 6 biological replicates). Statistical analysis was performed using one‐way ANOVA. **P ≤ 0.01.

Since mtROS production has been demonstrated to activate the NLRP3 inflammasome 32, 33 and CASP1 activity was reported to control phagosomal acidification in response to Gram‐positive bacteria 34, we subsequently examined whether Ant A‐induced ROS production enhanced CASP1 activation, which might contribute to the reduced bacterial loads observed in *casp11* ^−/−^ macrophages. As shown in Figs 5C and EV3A, Ant A treatment did not increase either CASP1 or IL‐1β cleavage in WT and *casp11* ^−/−^ macrophages, indicating that ROS‐mediated killing of intracellular MRSA is independent of CASP1. In accordance with previous studies, which demonstrated increased IL‐8 production in response to oxidative stress 35, 36, we found significantly higher amounts of CXCL1/KC produced by WT macrophages when treated with Ant A (Fig EV3A). No difference in the production of IL‐1α or TNF was observed in response to Ant A (Fig EV3A). Furthermore, the inhibition of lysosomal acidification using bafilomycin A1 (Baf A1) led to a marked decrease in bacterial loads in WT as well as *casp11* ^−/−^ macrophages (Fig EV3B), indicating that the acidification of bacteria‐containing phagosomes is needed to allow MRSA survival in macrophages. Overall, these data suggest that bacterial co‐localization with mitochondria and mtROS contribute to improved clearance of MRSA from *casp11* ^−/−^ macrophages, but it remains unclear why mitochondria show increased association with phagosomes in *casp11* ^−/−^ cells.

### Inhibition of the actin cytoskeleton prevents the dissociation of MRSA‐containing phagosomes from mitochondria and leads to bacterial killing

**Figure 6:**
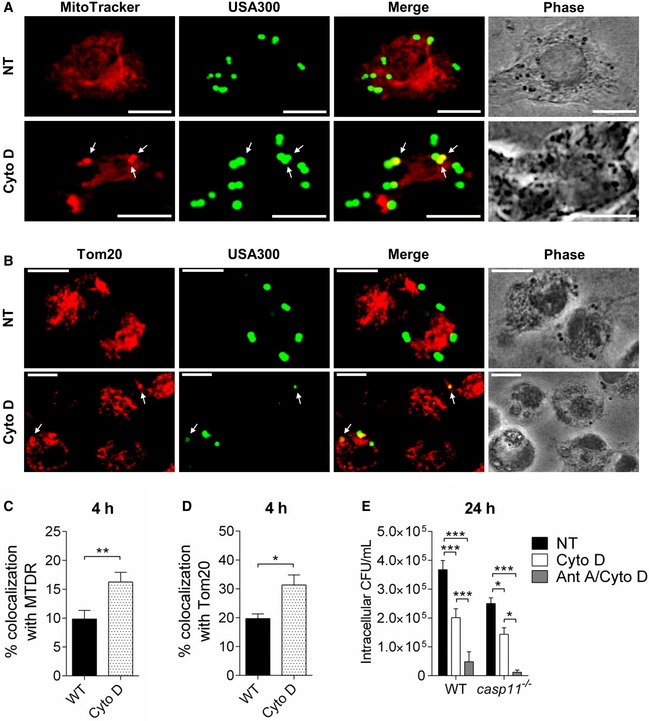
Cyto D improves ROS‐induced MRSA clearance in WT macrophages MitoTracker Deep Red immunofluorescence assay of MRSA‐infected WT BMDMs treated with Cyto D at 4 h post‐infection. White arrows indicate co‐localization of MRSA with MitoTracker.Tom20 immunofluorescence assay of MRSA‐infected WT BMDMs treated with Cyto D at 4 h post‐infection. White arrows indicate co‐localization of MRSA with Tom20.Quantification of MRSA co‐localized with MitoTracker Deep Red. Data represent mean ± SEM (*n* = 4 biological replicates). Statistical analysis was performed using two‐tailed Student's *t*‐test. **P ≤ 0.01.Quantification of MRSA co‐localized with Tom20. Data represent mean ± SEM (*n* = 3 biological replicates). Statistical analysis was performed using two‐tailed Student's *t*‐test. *P ≤ 0.05.Intracellular CFU of MRSA in WT and *casp11* ^−/−^ BMDMs treated with Cyto D or Cyto D + Ant A at 24 h post‐infection. Data represent mean ± SEM (*n* = 6 biological replicates). Statistical analysis was performed using two‐way ANOVA. *P ≤ 0.05, ***P ≤ 0.001, Data information: Scale bars, 10 μm.

**Figure 7:**
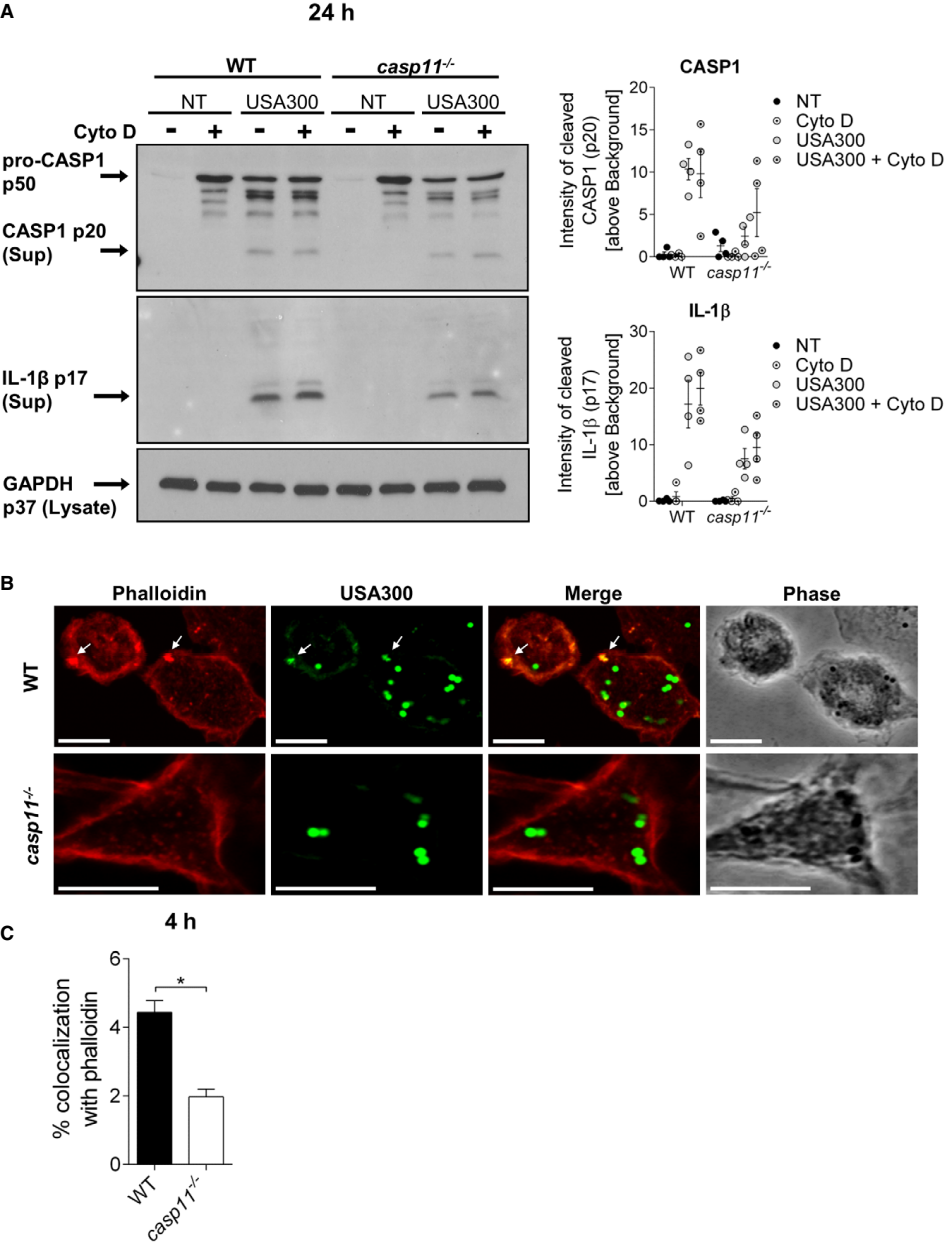
Cyto D improves MRSA clearance in WT macrophages independent of CASP1 Immunoblot analysis of cleaved CASP1 and IL‐1β in supernatants from WT and *casp11* ^−/−^ BMDMs treated with Cyto D at 24 h post‐infection. Densitometry analysis represents mean ± SEM (*n* = 4 biological replicates). Statistical analysis was performed using two‐way ANOVA. NT = no treatment.Rhodamine phalloidin immunofluorescence assay of MRSA‐infected WT and *casp11* ^−/−^ BMDMs at 4 h post‐infection.Quantification of MRSA co‐localized with rhodamine phalloidin. Data represent mean ± SEM (*n* = 3 biological replicates). Statistical analysis was performed using two‐tailed Student's *t*‐test. *P ≤ 0.05.Data information: Scale bars, 10 μm.

CASP11 has been shown to mediate cell migration 37 and to modulate actin dynamics in response to bacterial infection 8, 9. Since a higher percentage of MRSA is associated with mitochondria in *casp11* ^−/−^ macrophages, we hypothesize that the inhibition of actin polymerization would facilitate co‐localization of MRSA‐containing phagosomes with mitochondria in WT cells. We, therefore, treated WT macrophages with Cyto D subsequent to MRSA infection and examined them by either employing the fluorescent MitoTracker Deep Red dye or staining with an anti‐Tom20 antibody. In response to Cyto D, we found more bacteria co‐localized with MitoTracker or Tom20 compared to non‐treated cells (Fig 6A–D). Consistent with these findings, we also observed decreased bacterial loads at 24 h post‐infection in both Cyto D‐treated WT and *casp11* ^−/−^ macrophages (Fig 6E). Cyto D had no direct bactericidal activity against MRSA (Fig EV2A). Furthermore, the combination of Cyto D with Ant A led to an additive effect and a greater reduction in bacterial numbers as compared to Cyto D treatment alone (Fig 6E). While LDH release was comparable between non‐treated and Cyto D‐treated WT and *casp11* ^−/−^ macrophages infected with MRSA (Fig EV2C), there was no difference in extracellular bacterial loads (Fig EV2D). Similar to Ant A, testing for CASP1 and IL‐1β cleavage revealed no elevated inflammasome activity in response to Cyto D (Figs 7A and EV3C). Additionally, to test whether F‐actin assembles around the MRSA‐containing phagosome, we stained WT and *casp11* ^−/−^ macrophages with phalloidin. Interestingly, co‐localization of MRSA with phalloidin was mildly decreased in *casp11* ^−/−^ macrophages (Fig 7B and C). Together, these results suggest that impaired actin dynamics prevent the dissociation of mitochondria from internalized MRSA*,* thereby facilitating mtROS‐driven bacterial killing.

### mtROS production but not impaired autophagy contributes to MRSA clearance in *casp11* ^−/−^ macrophages

**Figure 8:**
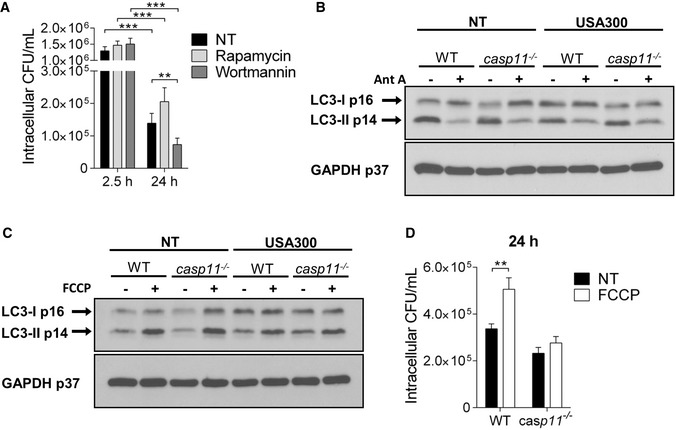
Compromised autophagy contributes to the clearance of intracellular MRSA Intracellular survival of MRSA in WT BMDMs treated with rapamycin or wortmannin. Data represent mean ± SEM (*n* = 5 biological replicates). Statistical analysis was performed using a linear mixed effects model. **P ≤ 0.01, ***P ≤ 0.001, NT = no treatment.Representative immunoblot of LC3‐II in cell lysates from WT and *casp11* ^−/−^ BMDMs treated with Ant A at 24 h post‐infection (*n* = 3 biological replicates).Representative immunoblot of LC3‐II in cell lysates from WT and *casp11* ^−/−^ BMDMs treated with FCCP at 24 h post‐infection (*n* = 3 biological replicates).Intracellular survival of MRSA in WT and *casp11* ^−/−^ BMDMs treated with FCCP. Data represent mean ± SEM (*n* = 4 biological replicates). Statistical analysis was performed using two‐way ANOVA. **P ≤ 0.01, NT = no treatment.

Autophagy/macroautophagy is a host cell defense mechanism that has been implicated in the degradation of various intracellular pathogens within double‐membrane structures called autophagosomes. Bacteria, including *S. aureus,* have developed strategies to escape from or even subvert the autophagic machinery 38. Since autophagosomes have been demonstrated to support *S. aureus* survival in HeLa cells 38, we examined the impact of pharmacological stimulation or inhibition of autophagy on intracellular MRSA survival in WT macrophages using rapamycin (mTOR inhibitor) or wortmannin (PI3K inhibitor), respectively. To circumvent the inhibitory effect of wortmannin on bacterial host cell entry, the treatment was given after phagocytosis of MRSA was completed. Consistent with previous findings, bacterial loads at 24 h post‐infection were mildly but not significantly increased in response to the autophagy‐stimulating drug rapamycin (Fig 8A). In contrast, wortmannin significantly reduced the intracellular burden of MRSA, suggesting that the suppression of autophagy by wortmannin 39, 40 among other effects facilitates MRSA clearance from murine macrophages. Rapamycin had minor bactericidal activity, whereas no effect could be observed for wortmannin (Fig EV2A). Notably, in addition to its function as inhibitor of mitochondrial respiration, Ant A has been reported to negatively affect the autophagy pathway 41. Since we found reduced bacterial loads of MRSA in Ant A‐treated *casp11* ^−/−^ macrophages, we questioned whether compromised autophagy in response to Ant A might contribute to improved bacterial clearance in the absence of CASP11. To elucidate the role of Ant A in the autophagic process during MRSA infection, we compared the levels of the autophagosomal marker protein LC3‐II in WT and *casp11* ^−/−^ macrophages either untreated or treated with Ant A via Western blot analysis. In accordance with previous findings 41, Ant A reduced the levels of LC3‐II in both non‐infected and MRSA‐infected WT and *casp11* ^−/−^ macrophages (Fig 8B). However, Ant A reduced bacterial numbers only in *casp11* ^−/−^ macrophages. Since *casp11* ^−/−^ macrophages exhibit increased co‐localization of MRSA with mitochondria (Fig 4A–D), this suggests that the stimulation of mtROS production by Ant A is the major cause for effective MRSA clearance, not the inhibition of autophagy.

In contrast, disruption of the mitochondrial membrane potential (MMP) through uncoupling agents, such as trifluoromethoxy carbonylcyanide phenylhydrazone (FCCP), has been demonstrated to induce both autophagy and selective mitophagy 42, 43. Correspondingly, FCCP treatment led to a significant increase in LC3‐II formation in WT and *casp11* ^−/−^ macrophages (Fig 8C). Yet, an increased bacterial burden due to the stimulation of autophagy via FCCP could be observed only in WT macrophages where MRSA‐containing phagosomes do not co‐localize with mitochondria (Fig 8D). Since FCCP had a direct bactericidal effect against MRSA (Fig EV2A), FCCP treatment was given post‐MRSA infection. Overall, while inhibiting autophagy can further augment the clearance of intracellular MRSA*,* we conclude that improved bactericidal activity of *casp11* ^−/−^ macrophages against MRSA is mainly attributed to localized mitochondria‐derived ROS in close proximity to bacteria‐containing phagosomes.

## Discussion

*Staphylococcus aureus* exhibits numerous virulence factors that have been reported to induce multiple inflammasomes. Specifically, the activation of the NLRP3 inflammasome has been shown in response to peptidoglycan or pore‐forming toxins such as Hla, leukocidin AB (Leu AB), and Panton–Valentine leukocidin (PVL), leading to cleavage of CASP1, IL‐1β/IL‐18 secretion, and pyroptosis 21, 22, 44, 45, 46. In addition, AIM2 and ASC have a protective role during acute central nervous infection with *S. aureus* 47. Although CASP11 is well known to mediate non‐canonical NLRP3 inflammasome signaling during Gram‐negative bacterial infection, a possible contribution of CASP11 in *S. aureus*‐induced inflammasome activation was not previously discussed. It is noteworthy that experimental systems investigating the role of NLRP3 during *S. aureus* infection often involved priming steps with either LPS or LTA 20, 44, 45, which are known to induce CASP11 expression through TLR4 or TLR2 signaling 6, 48. Furthermore, *L. monocytogenes* and *S. aureus* infection, as well as transfection of LTA, were recently demonstrated to activate CASP11 in murine macrophages 15. Therefore, it is plausible to conclude that CASP11 is needed for CASP1 processing and downstream events in response to *S. aureus*. Although in our study we did not observe cleavage of CASP11 in macrophages infected with MRSA, which might be attributed to differences between *S. aureus* strains, we found a significant reduction of cleaved CASP1 and IL‐1β in the absence of CASP11. Furthermore, we report a CASP11‐dependent release of activated CASP7 in cell culture supernatants. Cleavage of CASP7 during *S. aureus* infection has been described for keratinocytes and THP‐1 macrophages 49, 50, and in response to Hla 51. In the context of *L. monocytogenes* infection, CASP7 prevents plasma membrane damage caused by the pore‐forming toxin listeriolysin O (LLO) 51. Inflammasome‐dependent activation of CASP7 results in cleavage and inactivation of Poly (ADP‐ribose) polymerase 1 (PARP1) 52, 53, thereby allowing pro‐inflammatory NF‐κB target gene expression. *L. pneumophila*‐induced CASP7 activation facilitates phagosome–lysosome fusion 26. However, the outcome of CASP7 activity during *S. aureus* infection remains uncharacterized.

Since previous reports suggested a potential role of the necroptosis effector protein MLKL for IL‐1β secretion in human THP‐1 macrophages in response to MRSA 24, we analyzed processing of CASP1, IL‐1β, and CASP7 in MLKL‐deficient BMDMs. However, we did not observe any difference in the maturation or secretion of these proteins in murine macrophages lacking MLKL in response to MRSA. In contrast, increased bacterial burdens, CASP1 activation, and IL‐1β release were reported in skin biopsies from *mlkl* ^−/−^ mice upon subcutaneous MRSA infection 50. Therefore, it is possible that inflammasome signaling pathways activated in response to MRSA differ between cell types.

GSDMD cleaved by CASP11 or CASP1 promotes pyroptosis and IL‐1β secretion 11, 54, 55. Specifically, oligomerization of the N‐terminal GSDMD fragment leads to the formation of a plasma membrane pore allowing the release of cytosolic content 56, 57. Consistent with these findings, we demonstrate lower levels of cleaved CASP1, IL‐1β, and CASP7 in cell culture supernatants of MRSA‐infected *gsdmd* ^−/−^ macrophages compared to corresponding WT cells. Yet, in comparison to *casp11* ^−/−^ and *casp1* ^−/−^ macrophages, we found mildly increased amounts of IL‐1β and CASP7 cleavage products in *gsdmd* ^−/−^ macrophage supernatants. Although the immediate release of IL‐1β in response to inflammasome activation was shown to require GSDMD, there is also evidence for a GSDMD‐independent mechanism of IL‐1β secretion through phosphatidylinositol 4,5‐bisphosphate (PIP2) enriched plasma membrane domains 58. Since MRSA‐induced processing of IL‐1β and CASP7 still occurs in macrophages lacking GSDMD due to active CASP1, our results indicate that the secretion of their mature products in *gsdmd* ^−/−^ macrophages is indeed rather delayed but not prevented.

Intracellular survival of *S. aureus* has been demonstrated for various cell types, including macrophages, neutrophils, and endothelial cells 29, 59, 60, 61. Notably, multiple studies have reported that *S. aureus* exploits autophagy to either replicate within autophagosomes or scavenge nutrients 38, 62, 63, 64. Accordingly, we found increased survival of MRSA in response to the autophagy‐stimulating drugs rapamycin and FCCP, whereas the inhibition of autophagy via wortmannin or Ant A promoted bacterial clearance. The persistence of MRSA within host cells provides the bacterium with protection against antibiotics 61. When the antibiotic pressure is removed, MRSA escapes from host cells followed by extracellular replication and infection of neighboring cells. Hence, it is necessary to identify mechanisms that allow efficient clearance of intracellular MRSA. Recently, mitochondria‐derived ROS were shown to exert antimicrobial effects on intracellular MRSA 16, 31. There is emerging evidence that elevated production of mtROS drives the bactericidal activity of macrophages against intracellular bacterial pathogens such as *S. *Typhimurium, *L. monocytogenes*,* M. tuberculosis,* and *E. coli* 17, 19, 65, 66. Interestingly, Hla‐induced NLRP3 inflammasome activation promotes active dissociation of mitochondria from intracellular bacteria and thus contravenes mtROS‐driven eradication of MRSA 16. The recruitment and association of NLRP3 to and with mitochondria through direct interaction with mitochondrial cardiolipin 67, 68 indicates that mitochondria act as scaffolds to facilitate NLRP3 inflammasome activation. Although no difference in the bacterial burden of *S. aureus* between WT and *nlrp3* ^−/−^ mice was found in an earlier report 23, the inhibition of NLRP3 in human monocytes improves killing of intracellular MRSA due to increased bacterial association with mitochondria 16. Consequently, in the absence of Hla and NLRP3 activation, co‐localization of MRSA with mitochondria is more pronounced 16. Here, we show that the disengagement of MRSA‐containing phagosomes from mitochondria is also reduced in macrophages deficient of CASP11, allowing mtROS to contribute to bacterial clearance. Interestingly, we observed a decline in mitochondrial superoxide production in response to MRSA infection that was dependent on CASP11 expression in macrophages. In addition, elevated amounts of superoxide induced by Ant A exert bactericidal effects only in the absence of CASP11 where bacteria are found in the proximity of mitochondria. Notably, MRSA has been demonstrated to trigger mitochondria‐derived vesicle (MDV) formation in infected macrophages, leading to the delivery of the hydrogen peroxide‐generating enzyme superoxide dismutase‐2 (SOD2) to bacteria‐containing phagosomes 31. Since *casp11* ^−/−^ macrophages control bacterial loads more effectively in response to Ant A than corresponding WT cells, it is plausible to hypothesize that CASP11 might interfere with either MDV production or SOD2 delivery to the MRSA‐containing phagosome.

*Staphylococcus aureus* carries multiple genes encoding ROS‐detoxifying enzymes, including catalase and superoxide dismutase, to minimize the effects of oxidative stress 3, 69. Our results suggest that *S. aureus* defense mechanisms are less effective in macrophages lacking CASP11. The significant role of mtROS for MRSA eradication is further confirmed by the fact that the treatment of WT macrophages with Cyto D prevents MRSA dissociation from mitochondria, leading to improved bacterial clearance. Both actin filaments and microtubules have been implicated in the movement of mitochondria 70, 71. Considering that multiple reports describe actin dynamics to be defective in CASP11‐deficient cells 8, 13, 37, it is conceivable that CASP11 promotes the separation of MRSA‐containing phagosomes from mitochondria through the regulation of actin filaments.

In addition to mtROS, CASP1 was demonstrated to promote the killing of Hla‐deficient *S. aureus* by modulating phagosomal acidification 16, 34. However, others have shown that *S. aureus* resides and replicates in acidic compartments 72, 73, indicating that acidity does not reduce MRSA survival within macrophages. In our study, cleavage of CASP1 and subsequent IL‐1β release did not increase either in response to Ant A or Cyto D. In addition, there was no difference in the bacterial burden between WT and *casp1* ^−/−^ macrophages, and the inhibition of acidification using Baf A1 significantly reduced MRSA replication, indicating that acidification is required for MRSA persistence. We therefore conclude that CASP11 promotes MRSA persistence since it is required for the active dissociation of mitochondria away from the vicinity of MRSA‐containing vacuoles. Contrary to Gram‐positive bacteria, CASP11 promotes the clearance of Gram‐negative bacteria by promoting phagosome–lysosome fusion 8, 9, 13. Our data provide a new role for CASP11 in the pathogenesis of Gram‐positive bacteria.

## Materials and Methods

### Bacterial strains

*Staphylococcus aureus* strain USA300 was used in this study. USA300 complemented with a plasmid for green fluorescent protein (GFP) (BEI resources) was used in immunofluorescence experiments. All bacterial strains were grown in tryptic soy broth (TSB) at 37°C and 200 rpm overnight, and sub‐cultured the next day before use in experiments.

### Mice

C57BL/6 WT mice were obtained from the Jackson Laboratory (Bar Harbor, ME, USA). *Casp11* ^−/−^ mice were generously given by Dr. Yuan at Harvard Medical School, Boston, MA, USA 74. *Casp‐1* ^*−*/*−*/*Casp−11Tg*^ mice were kindly provided by Dr. Vishva Dixit at Genentech, San Francisco, CA, USA. *Gsdmd* ^−/−^, *mlkl* ^−/−^, and *nlrp3* ^−/−^ mice were a gift from Dr. Thirumala‐Devi Kanneganti at St. Jude Children's Research Hospital, Memphis, TN, USA. All mice were housed in a pathogen‐free facility, and experiments were conducted with approval from the Animal Care and Use Committee at the Ohio State University (Columbus, OH, USA).

### *In vivo* infection

For intratracheal infection, mice were anesthetized with Isoflurane and inoculated with 100 μl of PBS containing 2.5 × 10^8^ CFUs of MRSA USA300. To determine the bacterial load in organs, mice were sacrificed at 96 h post‐infection to collect lungs, for homogenization in PBS as previously described 9, 13, 75. Data are presented as CFUs per gram of organ tissue.

### Cell culture

For the generation of primary bone marrow‐derived macrophages (BMDMs) from mice, tibias and femurs were flushed with IMDM media (Thermo Fisher Scientific, 12440053) supplemented with 10% of heat inactivated fetal bovine serum (FBS, Thermo Fisher Scientific, 16000044), 50% L cell‐conditioned media, 0.6× MEM Non‐Essential Amino Acids (Thermo Fisher Scientific, 11140050), and 0.1% penicillin and streptomycin (Thermo Fisher Scientific, 15140122). Cells were cultivated at least 6 days at 37°C in a humidified atmosphere containing 5% CO_2_ as previously described 8, 9, 13, 76. THP‐1 cells either expressing Cas9 or co‐expressing *CASP4* ^−/−^, *CASP5* ^−/−^, *CASP4/5* ^−/−^, and *CASP1* ^−/−^ were maintained in RPMI 1640 media (Thermo Fisher Scientific, 22400105) supplemented with 10 % FBS and 0.1% penicillin and streptomycin. To induce the CRISPR system, cells were incubated with 1 μg/ml of doxycycline hyclate (Sigma, D9891) for 72 h and the knockout status was verified by immunoblotting 27. THP‐1 monocytes were differentiated into macrophages by 48‐h incubation with 25 nM phorbol 12‐myristate 13‐acetate (PMA, Sigma‐Aldrich, P8139) followed by 24‐h incubation in RPMI medium plus 10% FBS.

### *In vitro* infection of primary macrophages

Prior to infection, macrophages were cultivated in IMDM or RPMI media supplemented with 10% FBS. *In vitro* infections were performed with MOI 5:1 or MOI 20:1 as indicated, including centrifugation for 5 min at 200× *g* to synchronize the infection. For gentamicin protection assays and protein determination via Western blot analysis in cell culture lysates or supernatants, macrophages were infected for 1 h followed by two gentamicin treatments of cells (1 h followed by 30 min of incubation, respectively, with 100 and 40 μg/ml of gentamicin) to eliminate extracellular bacteria. Macrophages were then incubated with 10 μg/ml gentamicin until the end of the experiment in the presence or absence of Ant A (5 μM), Cyto D (2 μM), FCCP (5 μM), rapamycin (5 μg/ml), wortmannin (200 nM), NAC (3 mM), MitoQ (1 μM), or Baf A1 (100 nM) as indicated. For the determination of intracellular bacterial loads, macrophages were lysed at indicated time points using 0.1% Triton X‐100 (Fisher Scientific, BP151) in PBS, and CFUs of MRSA were determined in serial dilutions of the lysates using TSB agar plates incubated for 24 h.

### Confocal microscopy

Macrophages were cultured on glass coverslips in 24‐well plates and fixed with 4% paraformaldehyde for 30 min at indicated time points. Mitochondria of infected macrophages were stained for 15 min with 250 nM of MitoTracker™ Deep Red (Molecular Probes, M22426). MitoTracker™ Deep Red staining was pseudocolored in red. For permeabilization, cells were treated with 0.3% Triton X‐100 for 20 min followed by blocking with 5% goat serum (Thermo Fisher Scientific, 16210064) in PBS. Tom20 (Sigma‐Aldrich, WH0009804M1) was visualized using goat anti‐mouse IgG secondary antibody conjugated to Alexa Fluor^®^ 594 (Molecular Probes, A‐11032). Nuclei were counterstained with 1 μg/ml of Hoechst solution (Molecular Probes, 62249) for 15 min. Rhodamine phalloidin (Molecular Probes, R415) was used to stain F‐actin filaments for 20 min. Images were captured using a laser scanning confocal fluorescence microscope with a 60× objective (Olympus Fluoview FV10i) as previously described 8, 76.

### ELISA

Cytokines in cell culture supernatants were measured by R&D Systems DuoSet ELISA Development Systems (murine IL‐1α, DY400, murine IL‐1β, DY401, murine CXCL1/KC, DY453, murine TNF‐alpha, DY410, human IL‐1α, DY200, human IL‐1β, DY201, human IL‐8, DY208, human TNF‐alpha, DY210) according to the manufacturer's instructions.

### Immunoblotting

Protein extraction from macrophages was performed using TRIzol reagent according to the manufacturer's instructions. Briefly, after phase separation using chloroform, 100% ethanol was added to the interphase/phenol–chloroform layer to precipitate genomic DNA. Subsequently, the phenol–ethanol supernatant was mixed with isopropanol to isolate proteins. The Bradford method was used to determine protein concentrations in cell lysates. Equal amounts of protein or supernatants were separated by 13.5% SDS–PAGE and transferred to a polyvinylidene fluoride (PVDF) membrane. Membranes were incubated overnight with antibodies against CASP11 (Cell Signaling, 14340), murine CASP1 (Adipogen, AG‐20B‐0042‐C100), human cleaved CASP1 (Cell Signaling Technology, 4199), human CASP1 (Cell Signaling Technology, 2225), cleaved CASP7 (Cell Signaling Technology, 9491), human CASP4 (MBL, M0293), murine IL‐1β (R&D Systems, AF‐401‐NA), and GAPDH (Cell Signaling Technology, 2118). Corresponding secondary antibodies conjugated with horseradish peroxidase and in combination with enhanced chemiluminescence reagent were used to visualize protein bands. Densitometry analyses were performed by normalizing target protein bands to their respective loading control (GAPDH) using ImageJ software as previously described 8, 9.

### LDH assay

LDH release from macrophages infected with MRSA was measured using the CytoTox‐ONE Homogeneous Membrane Integrity Assay (Promega, G7891) according to the manufacturer's instructions. MRSA‐induced LDH release [%] = ((infected sample‐low control)/(high control‐low control))*100.

### Mitochondrial ROS assay

To determine mitochondrial superoxide production, macrophages were incubated for 30 min with 2 μM MitoSOX dye (Thermo Fisher Scientific, M36008) diluted in cell imaging solution (10 mM Hepes, 1 mg/ml BSA, 1 mg/ml Glucose, 1 mM MgCl_2_, 1.8 mM CaCl_2_ in PBS) at 37°C in a humidified atmosphere containing 5% CO_2_. Then, cells were washed with PBS and further incubated with cell imaging solution containing 10 μg/ml gentamicin. The fluorescence was read using a SpectraMax i3x microplate reader at 510/580 nm, and the cell number in each well was determined using a SpectraMax MiniMax 300 Imaging Cytometer. Fluorescence values were first normalized to the cell number in each well, and then, all conditions for each genotype were normalized to the WT non‐treated control group.

### Transmission electron microscopy

Macrophages were cultured in 2‐well Permanox Lab‐Tek chamber slides (Nunc, 177429) and fixed with 2.5% glutaraldehyde (Ted Pella, 18426) in 0.1 M phosphate buffer, pH 7.4 (Fisher Scientific, S369 and S373) containing 0.1 M sucrose (Fisher Scientific, S2‐500). Sample processing was performed by the Campus Microscopy & Imaging Facility at The Ohio State University as previously described 75. Images were taken using a FEI Tecnai G2 Spirit transmission electron microscope plus AMT camera system.

### Statistical analysis

Data were analyzed using GraphPad Prism 6.0. All figures display mean and standard error of the mean (SEM) from at least three independent experiments as indicated in the figure legends. Comparisons between groups were conducted with either Student's *t*‐test, one‐way or two‐way ANOVA, or linear mixed effects model (depending on the data structure) followed by Holm's adjustment for multiple comparisons as indicated. Adjusted *P* < 0.05 was considered statistically significant.

## Author contributions

Conceptualization, AOA, VP, and KK; Formal Analysis, KK; Investigation, KK, KD, SE, AB, KH, RH, MNKA, BK, XZ, MAG, and VP; Resources, AOA; Writing‐Original draft, KK; Writing‐Review & Editing, KK, AOA, KD, SE, AB, KH, AAK, RH, XZ, MAG, and VP; Project Administration, AOA and KK; Supervision, AOA; Funding Acquisition, AOA.

## Conflict of interest

The authors declare that they have no conflict of interest.

## Supporting information



Expanded View Figures PDF

Review Process File

## References

[1] Chambers HF (1997) Methicillin resistance in *Staphylococci*: molecular and biochemical basis and clinical implications. Clin Microbiol Rev 10: 781–791 9336672 10.1128/cmr.10.4.781 PMC172944

[2] Stapleton PD , Taylor PW (2002) Methicillin resistance in *Staphylococcus aureus*: mechanisms and modulation. Sci Prog 85(Pt 1): 57–72 11969119 10.3184/003685002783238870 PMC2065735

[3] Flannagan RS , Heit B , Heinrichs DE (2015) Antimicrobial mechanisms of macrophages and the immune evasion strategies of *Staphylococcus aureus* . Pathogens 4: 826–868 26633519 10.3390/pathogens4040826 PMC4693167

[4] Boswihi SS , Udo EE (2018) Methicillin‐resistant *Staphylococcus aureus*: an update on the epidemiology, treatment options and infection control. Curr Med Res Pract 8: 18–24

[5] Kayagaki N , Warming S , Lamkanfi M , Vande Walle L , Louie S , Dong J , Newton K , Qu Y , Liu J , Heldens S *et al* (2011) Non‐canonical inflammasome activation targets caspase‐11. Nature 479: 117–121 22002608 10.1038/nature10558

[6] Hagar JA , Powell DA , Aachoui Y , Ernst RK , Miao EA (2013) Cytoplasmic lps activates caspase‐11: implications in tlr4‐independent endotoxic shock. Science 341: 1250–1253 24031018 10.1126/science.1240988 PMC3931427

[7] Shi J , Zhao Y , Wang Y , Gao W , Ding J , Li P , Hu L , Shao F (2014) Inflammatory caspases are innate immune receptors for intracellular lps. Nature 514: 187–192 25119034 10.1038/nature13683

[8] Caution K , Gavrilin MA , Tazi M , Kanneganti A , Layman D , Hoque S , Krause K , Amer AO (2015) Caspase‐11 and caspase‐1 differentially modulate actin polymerization via rhoa and slingshot proteins to promote bacterial clearance. Sci Rep 5: 18479 26686473 10.1038/srep18479 PMC4685268

[9] Krause K , Caution K , Badr A , Hamilton K , Saleh A , Patel K , Seveau S , Hall‐Stoodley L , Hegazi R , Zhang X *et al* (2018) Casp4/caspase‐11 promotes autophagosome formation in response to bacterial infection. Autophagy 14: 1928–1942 30165781 10.1080/15548627.2018.1491494 PMC6152495

[10] Casson CN , Copenhaver AM , Zwack EE , Nguyen HT , Strowig T , Javdan B , Bradley WP , Fung TC , Flavell RA , Brodsky IE *et al* (2013) Caspase‐11 activation in response to bacterial secretion systems that access the host cytosol. PLoS Pathog 9: e1003400 23762026 10.1371/journal.ppat.1003400 PMC3675167

[11] Kayagaki N , Stowe IB , Lee BL , O'Rourke K , Anderson K , Warming S , Cuellar T , Haley B , Roose‐Girma M , Phung QT *et al* (2015) Caspase‐11 cleaves gasdermin d for non‐canonical inflammasome signalling. Nature 526: 666–671 26375259 10.1038/nature15541

[12] Wiggins KA , Parry AJ , Cassidy LD , Humphry M , Webster SJ , Goodall JC , Narita M , Clarke MCH (2019) Il‐1alpha cleavage by inflammatory caspases of the noncanonical inflammasome controls the senescence‐associated secretory phenotype. Aging Cell 18: e12946 30916891 10.1111/acel.12946 PMC6516163

[13] Akhter A , Caution K , Abu Khweek A , Tazi M , Abdulrahman BA , Abdelaziz DH , Voss OH , Doseff AI , Hassan H , Azad AK *et al* (2012) Caspase‐11 promotes the fusion of phagosomes harboring pathogenic bacteria with lysosomes by modulating actin polymerization. Immunity 37: 35–47 22658523 10.1016/j.immuni.2012.05.001 PMC3408798

[14] Aachoui Y , Leaf IA , Hagar JA , Fontana MF , Campos CG , Zak DE , Tan MH , Cotter PA , Vance RE , Aderem A *et al* (2013) Caspase‐11 protects against bacteria that escape the vacuole. Science 339: 975–978 23348507 10.1126/science.1230751 PMC3697099

[15] Hara H , Seregin SS , Yang D , Fukase K , Chamaillard M , Alnemri ES , Inohara N , Chen GY , Nunez G (2018) The nlrp6 inflammasome recognizes lipoteichoic acid and regulates gram‐positive pathogen infection. Cell 175: 1651–1664 30392956 10.1016/j.cell.2018.09.047 PMC6294477

[16] Cohen TS , Boland ML , Boland BB , Takahashi V , Tovchigrechko A , Lee Y , Wilde AD , Mazaitis MJ , Jones‐Nelson O , Tkaczyk C *et al* (2018) *Staphylococcus aureus* evades macrophage killing through nlrp3‐dependent effects on mitochondrial trafficking. Cell Rep 22: 2431–2441 29490278 10.1016/j.celrep.2018.02.027 PMC7160668

[17] West AP , Brodsky IE , Rahner C , Woo DK , Erdjument‐Bromage H , Tempst P , Walsh MC , Choi Y , Shadel GS , Ghosh S (2011) Tlr signalling augments macrophage bactericidal activity through mitochondrial ros. Nature 472: 476–480 21525932 10.1038/nature09973 PMC3460538

[18] Ariffin JK , das Gupta K , Kapetanovic R , Iyer A , Reid RC , Fairlie DP , Sweet MJ (2015) Histone deacetylase inhibitors promote mitochondrial reactive oxygen species production and bacterial clearance by human macrophages. Antimicrob Agents Chemother 60: 1521–1529 26711769 10.1128/AAC.01876-15 PMC4775968

[19] Roca FJ , Ramakrishnan L (2013) Tnf dually mediates resistance and susceptibility to mycobacteria via mitochondrial reactive oxygen species. Cell 153: 521–534 23582643 10.1016/j.cell.2013.03.022 PMC3790588

[20] Mariathasan S , Weiss DS , Newton K , McBride J , O'Rourke K , Roose‐Girma M , Lee WP , Weinrauch Y , Monack DM , Dixit VM (2006) Cryopyrin activates the inflammasome in response to toxins and atp. Nature 440: 228–232 16407890 10.1038/nature04515

[21] Craven RR , Gao X , Allen IC , Gris D , Bubeck Wardenburg J , McElvania‐Tekippe E , Ting JP , Duncan JA (2009) *Staphylococcus aureus* alpha‐hemolysin activates the nlrp3‐inflammasome in human and mouse monocytic cells. PLoS ONE 4: e7446 19826485 10.1371/journal.pone.0007446 PMC2758589

[22] Munoz‐Planillo R , Franchi L , Miller LS , Nunez G (2009) A critical role for hemolysins and bacterial lipoproteins in *Staphylococcus aureus*‐induced activation of the nlrp3 inflammasome. J Immunol 183: 3942–3948 19717510 10.4049/jimmunol.0900729 PMC2762867

[23] Kebaier C , Chamberland RR , Allen IC , Gao X , Broglie PM , Hall JD , Jania C , Doerschuk CM , Tilley SL , Duncan JA (2012) *Staphylococcus aureus* alpha‐hemolysin mediates virulence in a murine model of severe pneumonia through activation of the nlrp3 inflammasome. J Infect Dis 205: 807–817 22279123 10.1093/infdis/jir846 PMC3274379

[24] Kitur K , Parker D , Nieto P , Ahn DS , Cohen TS , Chung S , Wachtel S , Bueno S , Prince A (2015) Toxin‐induced necroptosis is a major mechanism of *Staphylococcus aureus* lung damage. PLoS Pathog 11: e1004820 25880560 10.1371/journal.ppat.1004820 PMC4399879

[25] Lamkanfi M , Kanneganti TD , Van Damme P , Vanden Berghe T , Vanoverberghe I , Vandekerckhove J , Vandenabeele P , Gevaert K , Nunez G (2008) Targeted peptidecentric proteomics reveals caspase‐7 as a substrate of the caspase‐1 inflammasomes. Mol Cell Proteomics 7: 2350–2363 18667412 10.1074/mcp.M800132-MCP200 PMC2596343

[26] Akhter A , Gavrilin MA , Frantz L , Washington S , Ditty C , Limoli D , Day C , Sarkar A , Newland C , Butchar J *et al* (2009) Caspase‐7 activation by the nlrc4/ipaf inflammasome restricts *Legionella pneumophila* infection. PLoS Pathog 5: e1000361 19343209 10.1371/journal.ppat.1000361 PMC2657210

[27] Baker PJ , Boucher D , Bierschenk D , Tebartz C , Whitney PG , D'Silva DB , Tanzer MC , Monteleone M , Robertson AA , Cooper MA *et al* (2015) Nlrp3 inflammasome activation downstream of cytoplasmic lps recognition by both caspase‐4 and caspase‐5. Eur J Immunol 45: 2918–2926 26173988 10.1002/eji.201545655

[28] Mueller NJ , Wilkinson RA , Fishman JA (2002) *Listeria monocytogenes* infection in caspase‐11‐deficient mice. Infect Immun 70: 2657–2664 11953408 10.1128/IAI.70.5.2657-2664.2002 PMC127953

[29] Flannagan RS , Heit B , Heinrichs DE (2016) Intracellular replication of *Staphylococcus aureus* in mature phagolysosomes in macrophages precedes host cell death, and bacterial escape and dissemination. Cell Microbiol 18: 514–535 26408990 10.1111/cmi.12527

[30] Jubrail J , Morris P , Bewley MA , Stoneham S , Johnston SA , Foster SJ , Peden AA , Read RC , Marriott HM , Dockrell DH (2016) Inability to sustain intraphagolysosomal killing of *Staphylococcus aureus* predisposes to bacterial persistence in macrophages. Cell Microbiol 18: 80–96 26248337 10.1111/cmi.12485 PMC4778410

[31] Abuaita BH , Schultz TL , O'Riordan MX (2018) Mitochondria‐derived vesicles deliver antimicrobial reactive oxygen species to control phagosome‐localized *Staphylococcus aureus* . Cell Host Microbe 24: 625–636 e5 30449314 10.1016/j.chom.2018.10.005 PMC7323595

[32] Zhou R , Yazdi AS , Menu P , Tschopp J (2011) A role for mitochondria in nlrp3 inflammasome activation. Nature 469: 221–225 21124315 10.1038/nature09663

[33] Heid ME , Keyel PA , Kamga C , Shiva S , Watkins SC , Salter RD (2013) Mitochondrial reactive oxygen species induces nlrp3‐dependent lysosomal damage and inflammasome activation. J Immunol 191: 5230–5238 24089192 10.4049/jimmunol.1301490 PMC3833073

[34] Sokolovska A , Becker CE , Ip WK , Rathinam VA , Brudner M , Paquette N , Tanne A , Vanaja SK , Moore KJ , Fitzgerald KA *et al* (2013) Activation of caspase‐1 by the nlrp3 inflammasome regulates the nadph oxidase nox2 to control phagosome function. Nat Immunol 14: 543–553 23644505 10.1038/ni.2595 PMC3708594

[35] DeForge LE , Preston AM , Takeuchi E , Kenney J , Boxer LA , Remick DG (1993) Regulation of interleukin 8 gene expression by oxidant stress. J Biol Chem 268: 25568–25576 8244994

[36] Remick DG , Villarete L (1996) Regulation of cytokine gene expression by reactive oxygen and reactive nitrogen intermediates. J Leukoc Biol 59: 471–475 8613691 10.1002/jlb.59.4.471

[37] Li J , Brieher WM , Scimone ML , Kang SJ , Zhu H , Yin H , von Andrian UH , Mitchison T , Yuan J (2007) Caspase‐11 regulates cell migration by promoting aip1‐cofilin‐mediated actin depolymerization. Nat Cell Biol 9: 276–286 17293856 10.1038/ncb1541

[38] Schnaith A , Kashkar H , Leggio SA , Addicks K , Kronke M , Krut O (2007) *Staphylococcus aureus* subvert autophagy for induction of caspase‐independent host cell death. J Biol Chem 282: 2695–2706 17135247 10.1074/jbc.M609784200

[39] Blommaart EF , Krause U , Schellens JP , Vreeling‐Sindelarova H , Meijer AJ (1997) The phosphatidylinositol 3‐kinase inhibitors wortmannin and ly294002 inhibit autophagy in isolated rat hepatocytes. Eur J Biochem 243: 240–246 9030745 10.1111/j.1432-1033.1997.0240a.x

[40] Wu YT , Tan HL , Shui G , Bauvy C , Huang Q , Wenk MR , Ong CN , Codogno P , Shen HM (2010) Dual role of 3‐methyladenine in modulation of autophagy via different temporal patterns of inhibition on class i and iii phosphoinositide 3‐kinase. J Biol Chem 285: 10850–10861 20123989 10.1074/jbc.M109.080796 PMC2856291

[41] Ma X , Jin M , Cai Y , Xia H , Long K , Liu J , Yu Q , Yuan J (2011) Mitochondrial electron transport chain complex iii is required for antimycin a to inhibit autophagy. Chem Biol 18: 1474–1481 22118681 10.1016/j.chembiol.2011.08.009 PMC3225892

[42] Berezhnov AV , Soutar MP , Fedotova EI , Frolova MS , Plun‐Favreau H , Zinchenko VP , Abramov AY (2016) Intracellular ph modulates autophagy and mitophagy. J Biol Chem 291: 8701–8708 26893374 10.1074/jbc.M115.691774 PMC4861439

[43] Lyamzaev KG , Tokarchuk AV , Panteleeva AA , Mulkidjanian AY , Skulachev VP , Chernyak BV (2018) Induction of autophagy by depolarization of mitochondria. Autophagy 14: 921–924 29458285 10.1080/15548627.2018.1436937 PMC6070013

[44] Holzinger D , Gieldon L , Mysore V , Nippe N , Taxman DJ , Duncan JA , Broglie PM , Marketon K , Austermann J , Vogl T *et al* (2012) *Staphylococcus aureus* panton‐valentine leukocidin induces an inflammatory response in human phagocytes via the nlrp3 inflammasome. J Leukoc Biol 92: 1069–1081 22892107 10.1189/jlb.0112014 PMC3476237

[45] Melehani JH , James DB , DuMont AL , Torres VJ , Duncan JA (2015) *Staphylococcus aureus* leukocidin a/b (lukab) kills human monocytes via host nlrp3 and asc when extracellular, but not intracellular. PLoS Pathog 11: e1004970 26069969 10.1371/journal.ppat.1004970 PMC4466499

[46] Muller S , Wolf AJ , Iliev ID , Berg BL , Underhill DM , Liu GY (2015) Poorly cross‐linked peptidoglycan in mrsa due to meca induction activates the inflammasome and exacerbates immunopathology. Cell Host Microbe 18: 604–612 26567511 10.1016/j.chom.2015.10.011 PMC4648675

[47] Hanamsagar R , Aldrich A , Kielian T (2014) Critical role for the aim2 inflammasome during acute cns bacterial infection. J Neurochem 129: 704–711 24484406 10.1111/jnc.12669 PMC3999210

[48] Cheng KT , Xiong S , Ye Z , Hong Z , Di A , Tsang KM , Gao X , An S , Mittal M , Vogel SM *et al* (2017) Caspase‐11‐mediated endothelial pyroptosis underlies endotoxemia‐induced lung injury. J Clin Invest 127: 4124–4135 28990935 10.1172/JCI94495 PMC5663346

[49] Soong G , Chun J , Parker D , Prince A (2012) *Staphylococcus aureus* activation of caspase 1/calpain signaling mediates invasion through human keratinocytes. J Infect Dis 205: 1571–1579 22457275 10.1093/infdis/jis244 PMC3415815

[50] Kitur K , Wachtel S , Brown A , Wickersham M , Paulino F , Penaloza HF , Soong G , Bueno S , Parker D , Prince A (2016) Necroptosis promotes *Staphylococcus aureus* clearance by inhibiting excessive inflammatory signaling. Cell Rep 16: 2219–2230 27524612 10.1016/j.celrep.2016.07.039 PMC5001919

[51] Cassidy SK , Hagar JA , Kanneganti TD , Franchi L , Nunez G , O'Riordan MX (2012) Membrane damage during *Listeria monocytogenes* infection triggers a caspase‐7 dependent cytoprotective response. PLoS Pathog 8: e1002628 22807671 10.1371/journal.ppat.1002628 PMC3395620

[52] Erener S , Petrilli V , Kassner I , Minotti R , Castillo R , Santoro R , Hassa PO , Tschopp J , Hottiger MO (2012) Inflammasome‐activated caspase 7 cleaves parp1 to enhance the expression of a subset of nf‐kappab target genes. Mol Cell 46: 200–211 22464733 10.1016/j.molcel.2012.02.016

[53] Bast A , Krause K , Schmidt IH , Pudla M , Brakopp S , Hopf V , Breitbach K , Steinmetz I (2014) Caspase‐1‐dependent and ‐independent cell death pathways in burkholderia pseudomallei infection of macrophages. PLoS Pathog 10: e1003986 24626296 10.1371/journal.ppat.1003986 PMC3953413

[54] He WT , Wan H , Hu L , Chen P , Wang X , Huang Z , Yang ZH , Zhong CQ , Han J (2015) Gasdermin d is an executor of pyroptosis and required for interleukin‐1beta secretion. Cell Res 25: 1285–1298 26611636 10.1038/cr.2015.139 PMC4670995

[55] Evavold CL , Ruan J , Tan Y , Xia S , Wu H , Kagan JC (2018) The pore‐forming protein gasdermin d regulates interleukin‐1 secretion from living macrophages. Immunity 48: 35–44 e6 29195811 10.1016/j.immuni.2017.11.013 PMC5773350

[56] Liu X , Zhang Z , Ruan J , Pan Y , Magupalli VG , Wu H , Lieberman J (2016) Inflammasome‐activated gasdermin d causes pyroptosis by forming membrane pores. Nature 535: 153–158 27383986 10.1038/nature18629 PMC5539988

[57] Sborgi L , Ruhl S , Mulvihill E , Pipercevic J , Heilig R , Stahlberg H , Farady CJ , Muller DJ , Broz P , Hiller S (2016) Gsdmd membrane pore formation constitutes the mechanism of pyroptotic cell death. EMBO J 35: 1766–1778 27418190 10.15252/embj.201694696 PMC5010048

[58] Monteleone M , Stanley AC , Chen KW , Brown DL , Bezbradica JS , von Pein JB , Holley CL , Boucher D , Shakespear MR , Kapetanovic R *et al* (2018) Interleukin‐1beta maturation triggers its relocation to the plasma membrane for gasdermin‐d‐dependent and ‐independent secretion. Cell Rep 24: 1425–1433 30089254 10.1016/j.celrep.2018.07.027

[59] Gresham HD , Lowrance JH , Caver TE , Wilson BS , Cheung AL , Lindberg FP (2000) Survival of *Staphylococcus aureus* inside neutrophils contributes to infection. J Immunol 164: 3713–3722 10725730 10.4049/jimmunol.164.7.3713

[60] Kubica M , Guzik K , Koziel J , Zarebski M , Richter W , Gajkowska B , Golda A , Maciag‐Gudowska A , Brix K , Shaw L *et al* (2008) A potential new pathway for *Staphylococcus aureus* dissemination: the silent survival of *Staphylococcus aureus* phagocytosed by human monocyte‐derived macrophages. PLoS ONE 3: e1409 18183290 10.1371/journal.pone.0001409 PMC2169301

[61] Rollin G , Tan X , Tros F , Dupuis M , Nassif X , Charbit A , Coureuil M (2017) Intracellular survival of *Staphylococcus aureus* in endothelial cells: a matter of growth or persistence. Front Microbiol 8: 1354 28769913 10.3389/fmicb.2017.01354 PMC5515828

[62] Mestre MB , Colombo MI (2012) *Staphylococcus aureus* promotes autophagy by decreasing intracellular camp levels. Autophagy 8: 1865–1867 23047465 10.4161/auto.22161 PMC3541307

[63] O'Keeffe KM , Wilk MM , Leech JM , Murphy AG , Laabei M , Monk IR , Massey RC , Lindsay JA , Foster TJ , Geoghegan JA *et al* (2015) Manipulation of autophagy in phagocytes facilitates *Staphylococcus aureus* bloodstream infection. Infect Immun 83: 3445–3457 26099586 10.1128/IAI.00358-15 PMC4534639

[64] Bravo‐Santano N , Ellis JK , Mateos LM , Calle Y , Keun HC , Behrends V , Letek M (2018) Intracellular *Staphylococcus aureus* modulates host central carbon metabolism to activate autophagy. mSphere 3: e00374–18 30089650 10.1128/mSphere.00374-18 PMC6083095

[65] Sonoda J , Laganiere J , Mehl IR , Barish GD , Chong LW , Li X , Scheffler IE , Mock DC , Bataille AR , Robert F *et al* (2007) Nuclear receptor err alpha and coactivator pgc‐1 beta are effectors of ifn‐gamma‐induced host defense. Genes Dev 21: 1909–1920 17671090 10.1101/gad.1553007 PMC1935029

[66] Garaude J , Acin‐Perez R , Martinez‐Cano S , Enamorado M , Ugolini M , Nistal‐Villan E , Hervas‐Stubbs S , Pelegrin P , Sander LE , Enriquez JA *et al* (2016) Mitochondrial respiratory‐chain adaptations in macrophages contribute to antibacterial host defense. Nat Immunol 17: 1037–1045 27348412 10.1038/ni.3509 PMC4994870

[67] Iyer SS , He Q , Janczy JR , Elliott EI , Zhong Z , Olivier AK , Sadler JJ , Knepper‐Adrian V , Han R , Qiao L *et al* (2013) Mitochondrial cardiolipin is required for nlrp3 inflammasome activation. Immunity 39: 311–323 23954133 10.1016/j.immuni.2013.08.001 PMC3779285

[68] Elliott EI , Miller AN , Banoth B , Iyer SS , Stotland A , Weiss JP , Gottlieb RA , Sutterwala FS , Cassel SL (2018) Cutting edge: mitochondrial assembly of the nlrp3 inflammasome complex is initiated at priming. J Immunol 200: 3047–3052 29602772 10.4049/jimmunol.1701723 PMC5916517

[69] Gaupp R , Ledala N , Somerville GA (2012) Staphylococcal response to oxidative stress. Front Cell Infect Microbiol 2: 33 22919625 10.3389/fcimb.2012.00033 PMC3417528

[70] Ligon LA , Steward O (2000) Role of microtubules and actin filaments in the movement of mitochondria in the axons and dendrites of cultured hippocampal neurons. J Comp Neurol 427: 351–361 11054698 10.1002/1096-9861(20001120)427:3<351::aid-cne3>3.0.co;2-r

[71] Hollenbeck PJ , Saxton WM (2005) The axonal transport of mitochondria. J Cell Sci 118(Pt 23): 5411–5419 16306220 10.1242/jcs.02745 PMC1533994

[72] Tranchemontagne ZR , Camire RB , O'Donnell VJ , Baugh J , Burkholder KM (2016) *Staphylococcus aureus* strain usa300 perturbs acquisition of lysosomal enzymes and requires phagosomal acidification for survival inside macrophages. Infect Immun 84: 241–253 26502911 10.1128/IAI.00704-15 PMC4694005

[73] Lacoma A , Cano V , Moranta D , Regueiro V , Dominguez‐Villanueva D , Laabei M , Gonzalez‐Nicolau M , Ausina V , Prat C , Bengoechea JA (2017) Investigating intracellular persistence of *Staphylococcus aureus* within a murine alveolar macrophage cell line. Virulence 8: 1761–1775 28762868 10.1080/21505594.2017.1361089 PMC5810471

[74] Wang S , Miura M , Jung YK , Zhu H , Li E , Yuan J (1998) Murine caspase‐11, an ice‐interacting protease, is essential for the activation of ice. Cell 92: 501–509 9491891 10.1016/s0092-8674(00)80943-5

[75] Abdulrahman BA , Khweek AA , Akhter A , Caution K , Kotrange S , Abdelaziz DH , Newland C , Rosales‐Reyes R , Kopp B , McCoy K *et al* (2011) Autophagy stimulation by rapamycin suppresses lung inflammation and infection by *Burkholderia cenocepacia* in a model of cystic fibrosis. Autophagy 7: 1359–1370 21997369 10.4161/auto.7.11.17660 PMC3359483

[76] Abdulrahman BA , Khweek AA , Akhter A , Caution K , Tazi M , Hassan H , Zhang Y , Rowland PD , Malhotra S , Aeffner F *et al* (2013) Depletion of the ubiquitin‐binding adaptor molecule sqstm1/p62 from macrophages harboring cftr deltaf508 mutation improves the delivery of *Burkholderia cenocepacia* to the autophagic machinery. J Biol Chem 288: 2049–2058 23148214 10.1074/jbc.M112.411728 PMC3548511

