# Supplementary material for: Caspase‐11 counteracts mitochondrial ROS‐mediated clearance of Staphylococcus aureus in macrophages
Source: EMBO Rep. 2019 Oct 21;20(12):e48109. doi: 10.15252/embr.201948109 (PMC6893291; doi:10.15252/embr.201948109)
Supplement: Supplementary file 1 — Expanded View Figures PDF [file EMBR-20-e48109-s001.pdf]

Expanded View Figures

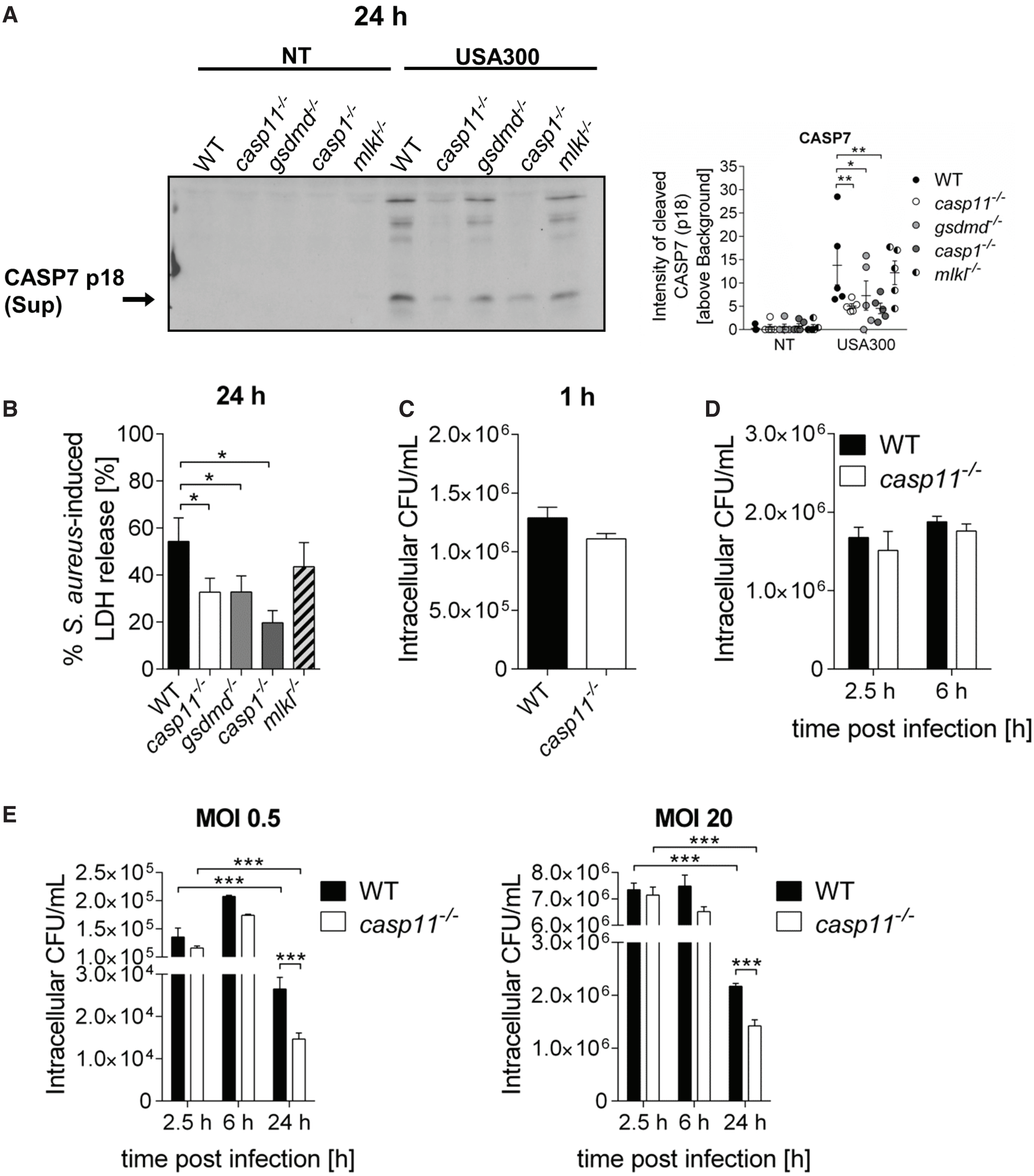

Figure EV1.

 © 2019 The Authors

Figure EV1. CASP11 promotes MRSA-induced inflammasome activation and MRSA persistence in macrophages.

A Immunoblot analysis of cleaved CASP7 in supernatants from WT, *casp11*$^{-/-}$, *gsdmd*$^{-/-}$, *casp1*$^{-/-}$, and *mlkl*$^{-/-}$ BMDMs infected with MRSA (MOI 20:1) at 24 h post-infection. Densitometry analysis represents mean ± SEM (n = 5 biological replicates). Statistical analysis was performed using two-way ANOVA. *$P \leq 0.05$, **$P \leq 0.01$, NT = no treatment.

B MRSA-induced LDH released in supernatants from WT, *casp11*$^{-/-}$, *gsdmd*$^{-/-}$, *casp1*$^{-/-}$, and *mlkl*$^{-/-}$ BMDMs at 24 h post-infection (MOI 20:1). Data represent mean ± SEM (n = 8 biological replicates). Statistical analysis was performed using one-way ANOVA. *$P \leq 0.05$.

C Intracellular CFU of MRSA (MOI 5:1) in WT and *casp11*$^{-/-}$ BMDMs at 1 h post-infection. Data represent mean ± SEM (n = 3 biological replicates). Statistical analysis was performed using two-tailed Student's t-test.

D Intracellular survival of MRSA (MOI 5:1) in WT and *casp11*$^{-/-}$ BMDMs. Data represent mean ± SEM (n = 5 biological replicates). Statistical analysis was performed using a linear mixed effects model.

E Intracellular survival of MRSA (MOI 0.5:1 and 20:1) in WT and *casp11*$^{-/-}$ BMDMs. Data represent mean ± SEM (n = 3 biological replicates). Statistical analysis was performed using a linear mixed effects model. ***$P \leq 0.001$.

© 2019 The Authors

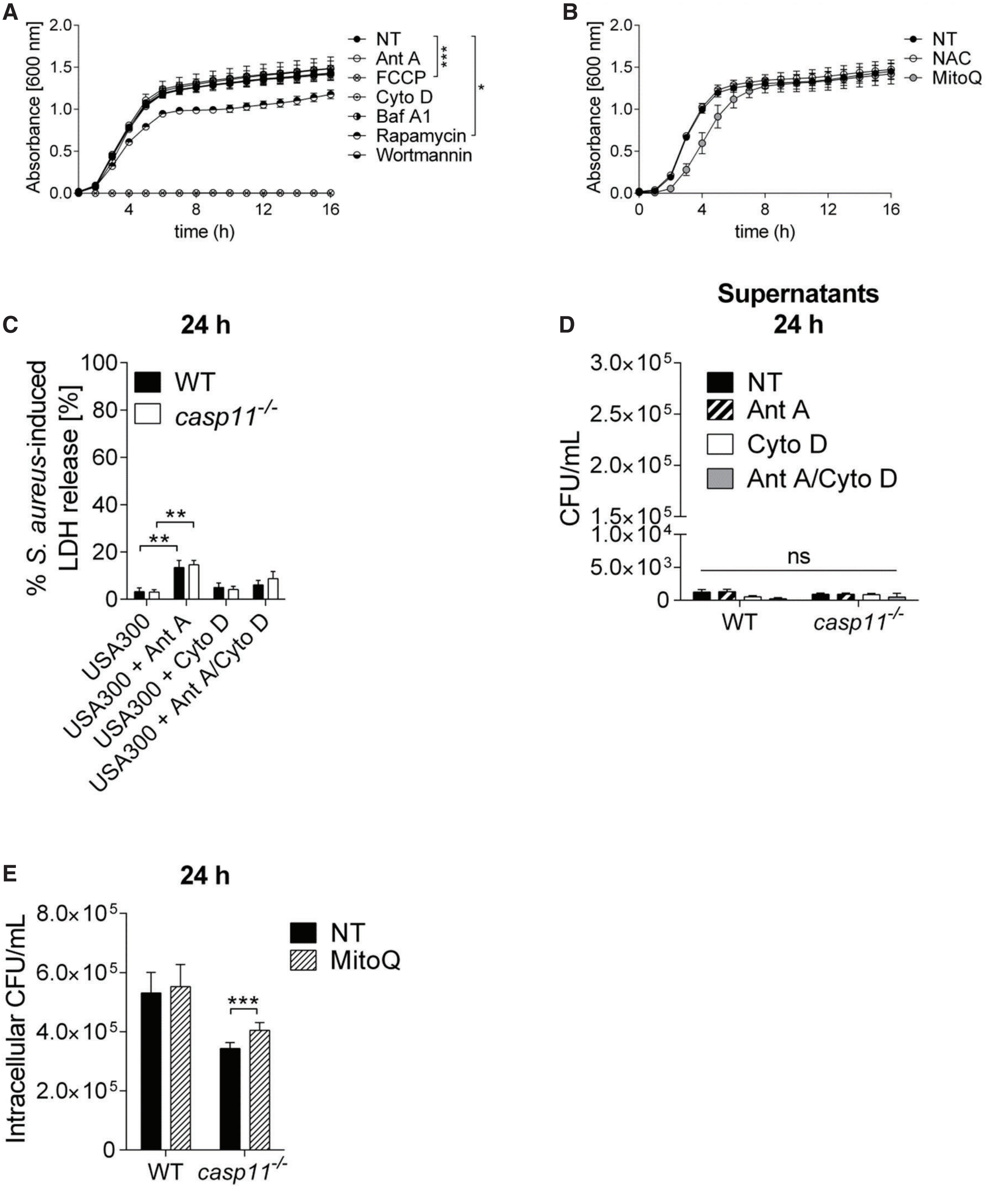

Figure EV2. Role of inhibitors affecting mtROS production, autophagy, and F-actin polymerization on MRSA growth and macrophage viability.

A MRSA growth in TSB in the presence of Ant A, FCCP, Cyto D, BafA1, rapamycin, and wortmannin (n = 3 biological replicates). Statistical analysis was performed using two-way ANOVA. *$P \leq 0.05$, ***$P \leq 0.001$, NT = no treatment.

B MRSA growth in TSB in the presence of NAC and MitoQ (n = 9 biological replicates). Statistical analysis was performed using two-way ANOVA. NT = no treatment.

C MRSA-induced LDH release in supernatants from WT and *casp11*$^{-/-}$ BMDMs (MOI 5:1) treated with Ant A or Cyto D at 24 h post-infection (n = 8 biological replicates). Statistical analysis was performed using two-way ANOVA. **$P \leq 0.01$.

D Extracellular growth of MRSA in cell culture supernatants from WT and *casp11*$^{-/-}$ BMDMs (MOI 5:1) treated with Ant A or Cyto D at 24 h post-infection (n = 8 biological replicates). Statistical analysis was performed using two-way ANOVA. NT = no treatment.

E Intracellular CFU of MRSA in WT and *casp11*$^{-/-}$ macrophages treated with MitoQ at 24 h post-infection (MOI 5:1). Data represent mean ± SEM (n = 8 biological replicates). Statistical analysis was performed using two-way ANOVA. ***$P \leq 0.001$, NT = no treatment.

© 2019 The Authors

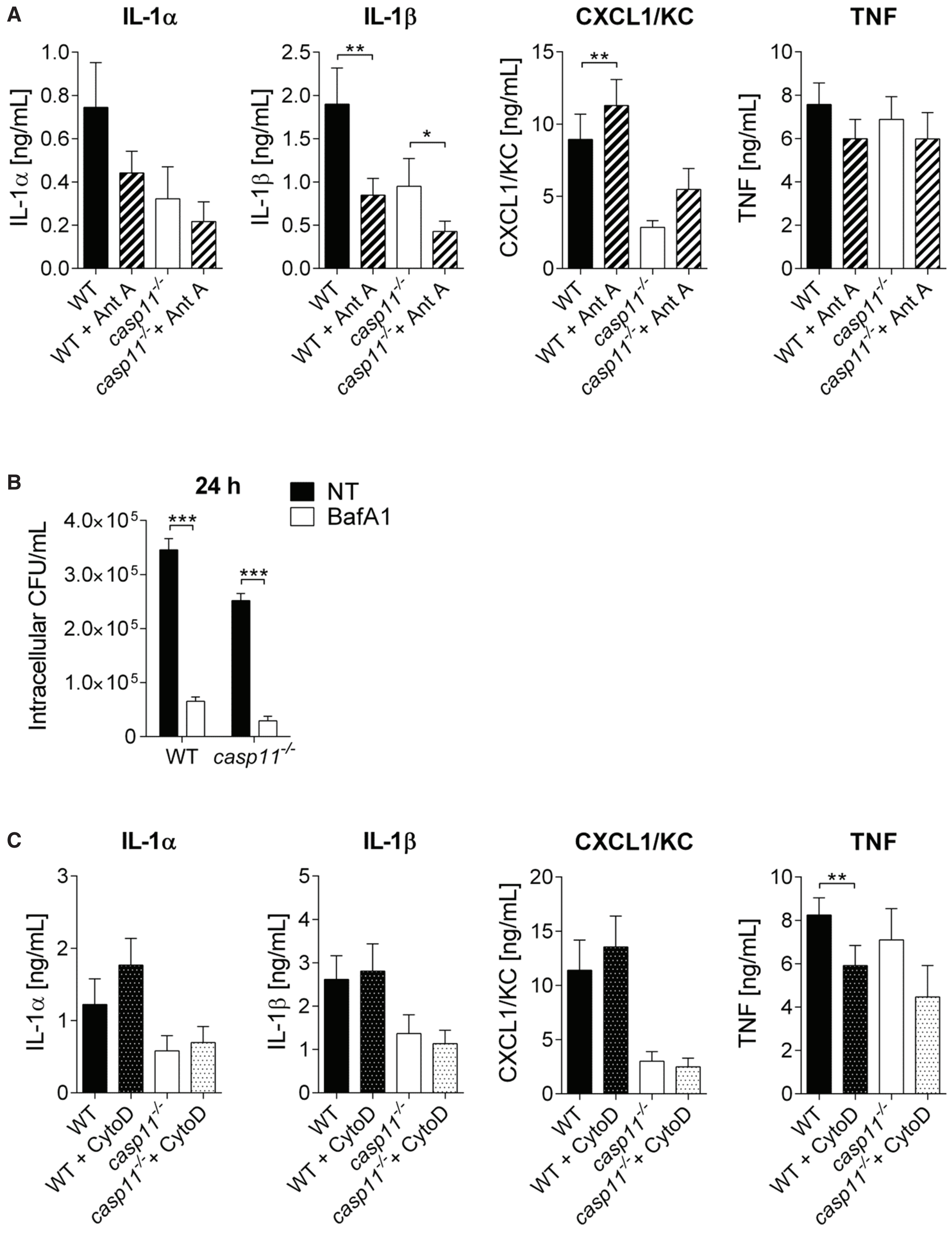
A
IL-1α
IL-1β
CXCL1/KC
TNF
IL-1α [ng/mL]
IL-1β [ng/mL]
CXCL1/KC [ng/mL]
TNF [ng/mL]
WT
WT + Ant A
casp11-/-
casp11-/- + Ant A
B
24 h
NT
BafA1
Intracellular CFU/mL
WT
casp11-/-
C
IL-1α
IL-1β
CXCL1/KC
TNF
WT + CytoD
casp11-/- + CytoD

Figure EV3.

Figure EV3. Effects of Ant A, Baf A1, and Cyto D during MRSA infection in macrophages.

A Cytokine release from MRSA-infected WT and *casp11*$^{-/-}$ macrophages treated with Ant A at 24 h post-infection (MOI 20:1). Data represent mean ± SEM (n = 8 biological replicates). Statistical analysis was performed using one-way ANOVA. *$P \leq 0.05$, **$P \leq 0.01$.

B Intracellular CFU of MRSA in WT and *casp11*$^{-/-}$ macrophages treated with BafA1 at 24 h post-infection (MOI 5:1). Data represent mean ± SEM (n = 3 biological replicates). Statistical analysis was performed using two-way ANOVA. ***$P \leq 0.001$, NT = no treatment.

C Cytokine release from MRSA-infected WT and *casp11*$^{-/-}$ macrophages treated with Cyto D at 24 h post-infection (MOI 20:1). Data represent mean ± SEM (n = 6 biological replicates). Statistical analysis was performed using one-way ANOVA. **$P \leq 0.01$.

© 2019 The Authors
